# Cyclopropane Hydrocarbons from the Springtail *Vertagopus sarekensis—*A New Class of Cuticular Lipids from Arthropods

**DOI:** 10.1021/acs.jnatprod.3c00789

**Published:** 2023-11-13

**Authors:** Anton Möllerke, Jan Bello, Hans Petter Leinaas, Stefan Schulz

**Affiliations:** † TU Braunschweig, Institute of Organic Chemistry, Hagenring 30, 38106 Braunschweig, Germany; § University of Oslo, Blindernveien, 310371 Oslo, Norway

## Abstract

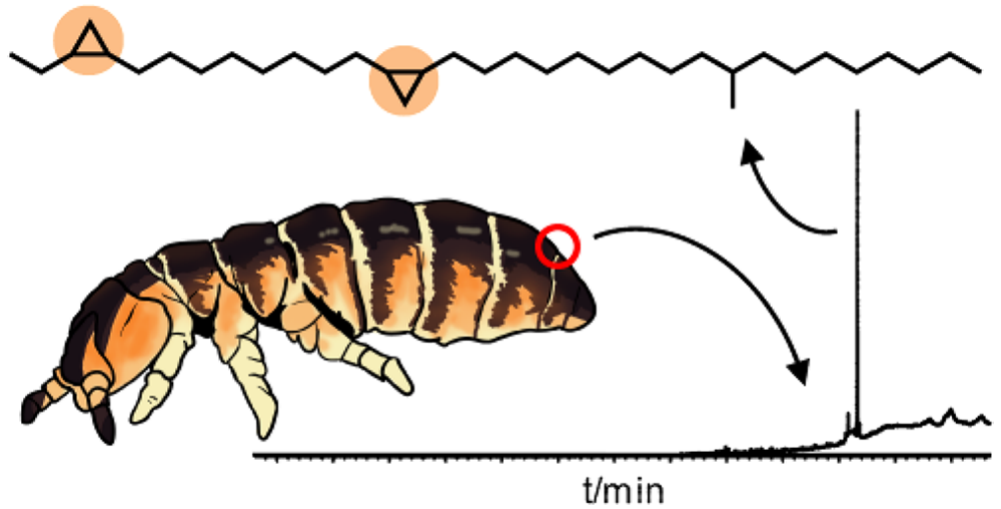

The epicuticle of insects is usually coated with a complex mixture of hydrocarbons, primarily straight-chain and methyl-branched alkanes and alkenes. We were interested in whether springtails (Collembola), a sister class of the insects, also use such compounds. We focused here on *Vertagopus sarekensis*, an abundant Isotomidae species in European high alpine regions, exhibiting coordinated group behavior and migration. This coordination, suggesting chemical communication, made the species interesting for our study on epicuticular hydrocarbons in springtails with different degrees of group behavior. We isolated a single hydrocarbon from its surface, which is the major epicuticular lipid. The structure was deduced by NMR analysis and GC/MS including derivatization. Total synthesis confirmed the structure as *cis*,*cis*-3,4,13,14-bismethylene-24-methyldotriacontane (**4**, sarekensane). The GC/MS analyses of some other cyclopropane hydrocarbons also synthesized showed the close similarity of both mass spectra and gas chromatographic retention indices of alkenes and cyclopropanes. Therefore, analyses of cuticular alkenes must be performed with appropriate derivatization to distinguish these two types of cuticular hydrocarbons. Sarekensane (**4**) is the first nonterpenoid cuticular hydrocarbon from Collembola that is biosynthesized via the fatty acid pathway, as are insect hydrocarbons, and contains unprecedented cyclopropane rings in the chain, not previously reported from arthropods.

The Collembola form a sister class to the Insecta, which diverged from the Insecta 400 mya. Their chemistry is poorly characterized compared to insects, but their unique chemical defense using compounds such as pyridopyrazines^1,2^ or sigillins^3,4^ has occasionally been investigated. As is probably the case in all arthropods, the collembolan cuticle is covered by a thin lipid film that is thought to prevent desiccation and may have additional functions such as signaling and protection against microbial attack, as has been shown in insects. In addition, the cuticle is often more hydrophobic than that of insects, even superhydrophobic, allowing many species to float on water.^5−8^

Insects usually use a complex mixture of many compounds derived from the fatty acid biosynthetic pathway^9^ as the epicuticular layer, consisting mainly of *n*- and methyl-branched alkanes and alkenes, but it may also contain other components such as long-chain esters, aldehydes, alcohols, or bisalkyltetrahydrofurans.^10^ This pathway is believed to be basal, developing before arthropod colonization of land.^11^ The few Collembola studied so far with respect to their cuticular chemistry, however, depend on a few major compounds of terpenoid origin, often of unusual structure. Examples are the [8]-terpene poduran (**1**) from *Podura aquatica*(12) and the branched [6^14^ + 2^1^]-terpene viaticene A from *Hypogastrura viatica* (**2**),^13^ while other species rely on conventional lycopane (**3**), a [4^1^ + 4^1^]-terpene, and unsaturated derivatives (Chart 1).^7,14^ Fatty acid-derived hydrocarbons have only been reported as minor constituents of whole-body extracts of *Tetrodontophora bielanensis*,^14^ although a more recent report on this species did not confirm their presence on the cuticle.^7^

**Chart 1:**
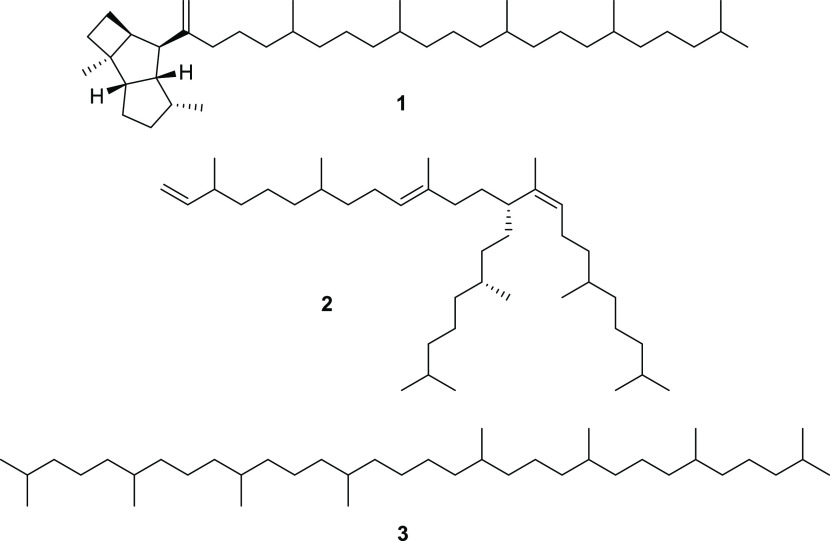
Epicuticular Lipids of Various Collembola

Although springtails are important members of soil ecosystems and contribute enormously to nutrient recycling, their chemistry has not been well studied.^15^ This is probably due to their small body size, difficult taxonomy, and often hidden lifestyle. In many cases, wild springtails cannot be collected in sufficient numbers, and laboratory cultivation is difficult and time consuming. Therefore, we have focused our research on species that occur locally in sufficient numbers to permit chemical analyses. One such species is *Vertagopus sarekensis*, which lives in the higher alpine regions of Europe under harsh conditions, feeding on algae and microorganisms found on mosses.^16^ It forms very large aggregations with coordinated group behavior and migration.^16^ This coordination, fairly common in some springtail families and suggesting chemical communication,^17^ made the species interesting for our study on epicuticular hydrocarbons in springtails with different degrees of group behavior.^13^

The analysis of insect cuticular hydrocarbons is well established using GC/MS and interpretation of mass spectra,^18^ gas chromatographic retention indices,^19,20^ as well as microderivatization with dimethyldisulfide for the localization of double bonds in alkenes.^21^ GC/MS allows the analysis of small amounts of complex mixtures of cuticular lipids, usually leading to meaningful results. Such an analysis of *V. sarekensis* revealed a single cuticular compound with a mass spectrum typical for an aliphatic diene with 35 carbons, probably of terpenoid origin based on the carbon number. Surprisingly, several attempts to perform dimethyldisulfide addition or ozonolysis failed, suggesting that this compound, which we term sarekensane, is structurally distinct from those commonly encountered as cuticular hydrocarbons. Isolation, spectroscopic analysis, and total synthesis revealed this compound to be a long-chain hydrocarbon containing cyclopropanes. In the following section, we describe the structural characterization and synthesis of this unique springtail cuticular component, which, unlike previously reported springtail hydrocarbons, is likely derived from the fatty acid biosynthetic pathway. In addition, the mass spectrometry of various analogs is described, which will hopefully be useful for further characterization of other compounds of this class and their differentiation from alkenes that show similar chromatographic and mass spectrometric behavior.

## Results and Discussion

### Structure Elucidation

**Figure 1:**
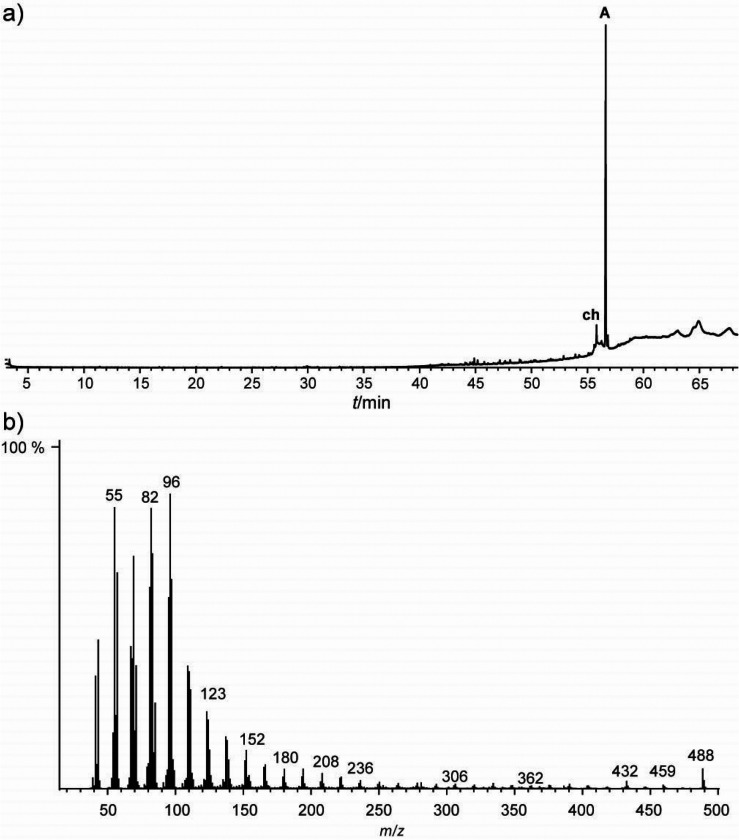
Total ion chromatogram (TIC) of a pentane extract of *V. sarekensis* (a), and mass spectrum of compound **A** (b); **ch**, cholesterol.

*Vertagopus sarekensis* were collected in the field where they occur in dense colonies. The GC/MS analysis of a pentane extract of the whole animals revealed the presence of only one major compound **A**, whose mass spectrum is shown in Figure 1. The mass spectrum showed the typical fragmentation pattern of an alkene with two double-bond equivalents. However, microreaction with dimethyl disulfide, which is commonly used to locate double-bond positions,^22^ or ozonolysis left the compound unchanged.

**Figure 2:**
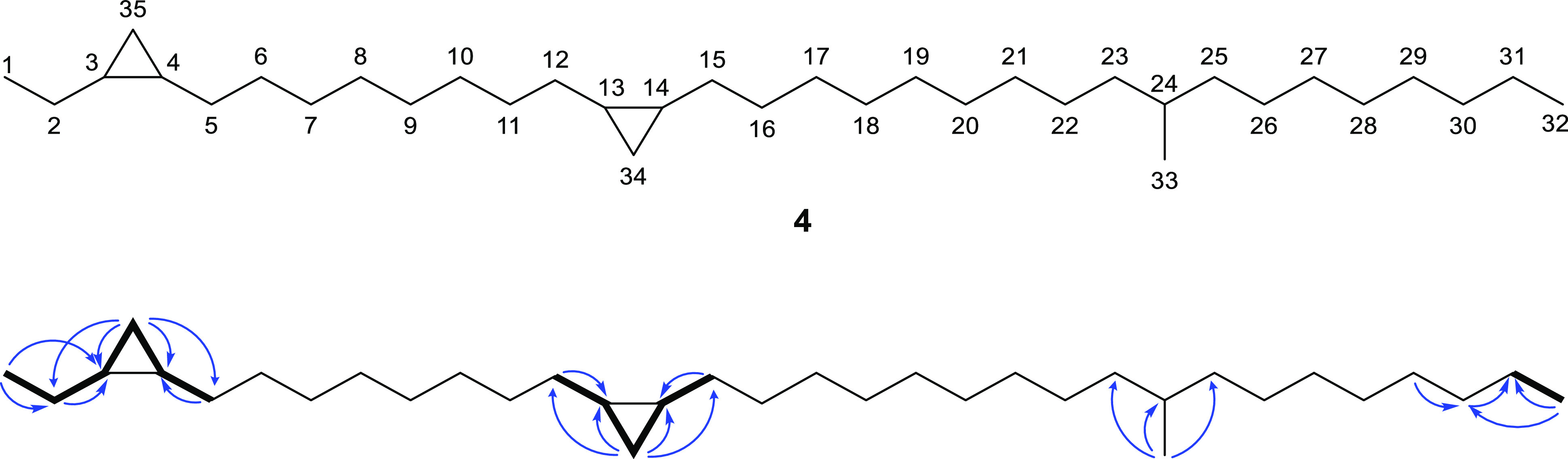
Major HMBC (blue arrows) and COSY (bold) NMR interactions of natural compound **A** (**4**).

Compound **A** (1.5 mg) was then isolated, and the ^1^H and ^13^C NMR data revealed the presence of two cyclopropane units. Two methylene units at 10.68 and 10.91 ppm correlated with the proton signal at −0.33 ppm, characteristic for cyclopropanes (Table 1). HMBC and COSY spectra also showed that both cyclopropane rings are disubstituted, one of them being substituted by an ethyl group (Figure 2). The other substituents appeared to be alkyl chains. The lack of correlation with other motifs than CH_2_ suggested an isolated position of the rings within a long alkane backbone. The HMBC data showed the presence of an additional isolated methyl branch at 19.7 ppm, which appeared as a doublet in the ^1^H spectrum at 0.84 ppm. The exact position of these three structural motifs on the straight C_32_ backbone could not be elucidated by NMR, due to their isolated nature, with the exception of the first cyclopropane unit, which is located at C-3/C-4.

**Table 1:** NMR Data (^1^H 500 MHz, ^13^C 125 MHz, CDCl_3_) of the Synthetic Material (**4**) and Natural Compound **A**

	synthetic **4**			natural compound **A**		
position	δ_C_, type	δ_H_ (*J* in Hz)	HMBC	δ_C_, type	δ_H_ (*J* in Hz)	HMBC
1	14.48, CH_3_	0.98, t (7.3)		14.48, CH_3_	0.98, t (7.3)	
2	21.94, CH_2_	1.45–1.33, m	1, 35	21.92, CH_2_	1.41–1.34, m	1, 35
		1.33–1.02, m			1.34–1.01, m	
3	17.72, CH	0.72–0.59, m	35	17.69, CH	0.68–0.59, m	35
4	15.98, CH	0.72–0.59, m	35	15.95, CH	0.68–0.59, m	35
5	28.65, CH_2_	1.45–1.33, m	35	28.62, CH_2_	1.41–1.34, m	35
		1.33–1.02, m			1.34–1.01, m	
6–11^a^	30.24, 29.73, 29.71, 29.39, 27.11, CH_2_			30.23, 29.73, 29.70, 29.37 27.09, CH_2_		
12/15	28.75, CH_2_	1.45–1.33, m	34	28.72, CH_2_	1.41–1.34, m	34
		1.33–1.02, m			1.34–1.01, m	
13	15.80, CH		34	15.78, CH	0.68–0.59, m	34
14	15.80, CH		34	15.78, CH	0.68–0.59, m	34
16–20^a^	30.24, 29.73, 29.71, 29.39, 27.11, CH_2_			30.23, 29.73, 29.70, 29.37 27.09, CH_2_		
21	30.25, CH_2_		1	30.23, CH_2_		1
22^a^	30.24, 29.73, 29.71, 29.39, 27.11, CH_2_			30.23, 29.73, 29.70, 29.37 27.09, CH_2_		
23/25	37.13, CH_2_	1.33–1.02, m	33	37.10, CH_2_	1.34–1.01, m	33
24	32.78, CH	1.45–1.33, m	33	32.75, CH	1.41–1.34, m	33
26	30.07, CH_2_			30.04, CH_2_	1.34–1.01, m	
27–29^a^	30.24, 29.73, 29.71, 29.39, 27.11, CH_2_			30.23, 29.73, 29.70, 29.37 27.09, CH_2_		
30	31.96, CH_2_	1.33–1.02, m	32	31.94, CH_2_	1.34–1.01, m	32
31	22.71, CH_2_	1.33–1.02, m		22.70, CH_2_	1.34–1.01, m	
32	14.13, CH_3_	0.88, t (7.0)		14.13, CH_3_	0.88, t (6.9)	
33	19.74, CH_3_	0.84, d (6.6)		19.73, CH_3_	0.83, d (6.6)	
34	10.70, CH_2_	–0.33, dd (9.5, 5.3), 0.59–0.53, m	12, 15	10.68, CH_2_	–0.33, dd (9.5, 5.3), 0.59–0.53, m	12/15
35	10.93, CH_2_	–0.33, dd (9.5, 5.3), 0.59–0.53, m	5	10.91, CH_2_	–0.33, dd (9.5, 5.3), 0.59–0.53, m	5

a Overlapping signals.

In order to identify the positions of the cyclopropyl groups, a microhydrogenation of **A** with Pd/C was performed, yielding a mixture of methyl-branched linear alkanes. As each ring can be opened in three different positions, giving either a methyl-branched or a CH_2_-extended alkane, nine different constitutional isomers were formed. The positions of the methyl groups in linear alkanes can be readily identified by EI-MS^18,23−25^ because they are indicated by an increased intensity of secondary cations obtained by cleavage next to the methyl group. Positional isomers of methyl-branched alkanes close to the chain end can be separated by GC on an apolar phase, while internal positional isomers coelute.^19,20^

**Figure 3:**
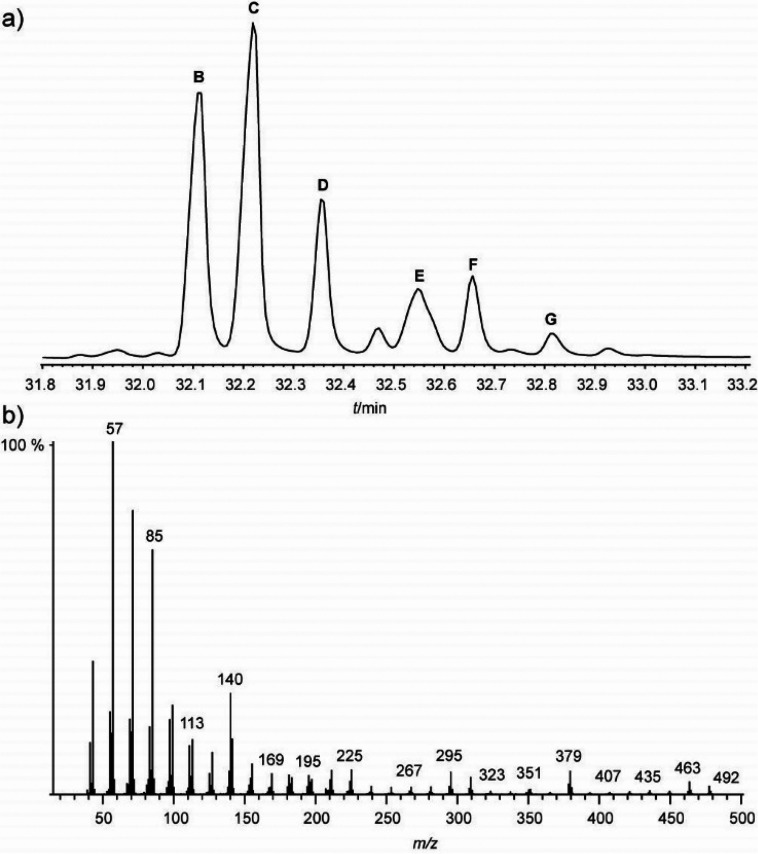
TIC of hydrogenated compound **A** (a), and mass spectrum of compound **C** (b). The peaks **B–G** are the hydrogenation products of **A**, indicated by a characteristic ion at *m/*z 477 [M – 15]^+^. The nonlabeled peaks are incomplete hydrogenation products or impurities.

**Figure 4:**
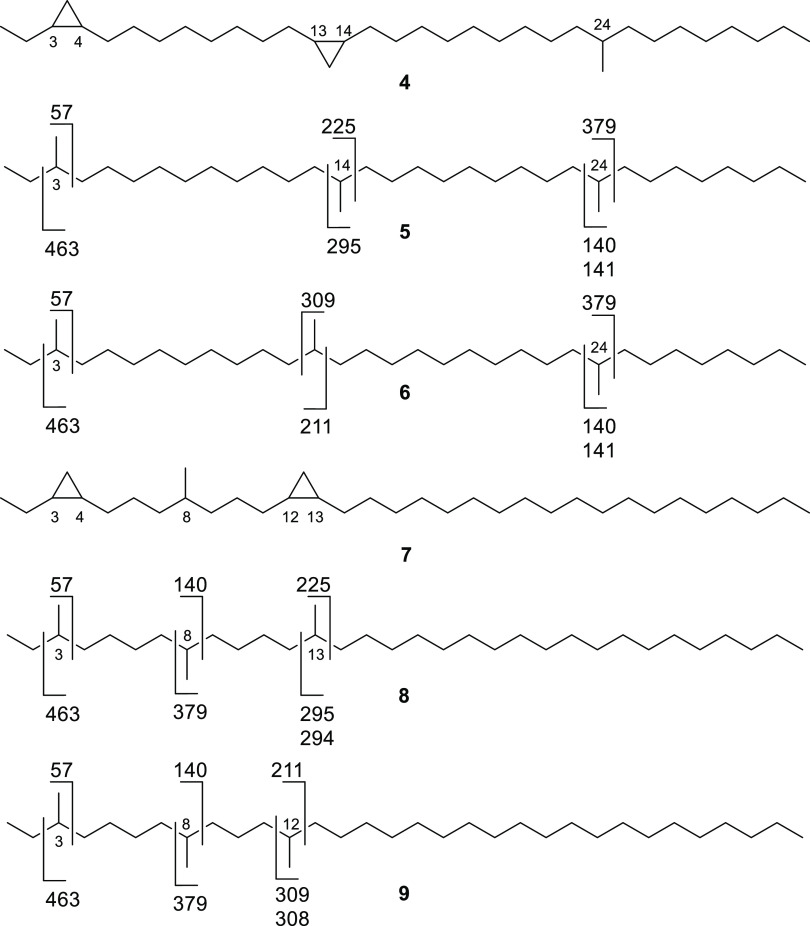
Possible structures (**4**, **7**) for natural compound **A** and mass spectrometric fragmentations of the respective hydrogenation products (**5**, **6**, **8**, **9**), derived from peak **C**. The mass spectrum of **C** is shown in Figure 3b. Compound **4** is the correct structure for **A**.

The GC/MS analysis of the hydrogenated sample (Figure 3a) showed six peaks (**B**–**G**) with an ion at *m*/*z* 477 [M – 15]^+^, characteristic for C_35_H_72_ alkanes. The mass spectrum of the last eluting compound **G** showed characteristic ions at *m*/*z* 140/141 and 378/379, consistent with 9-methyltetratriacontane. These ion pairs were present in all spectra **B**–**G**, confirming that the methyl group in compound **A** is found at C-9 or the ω-9 position. Compound **F** had an additional ion at *m*/*z* 463 [M – 29]^+^ together with stronger ions at *m*/*z* 56 and 70, indicating 3,25-dimethyltritriacontane, while the shift to *m*/*z* 449 [M – 43]^+^, 70, and 84 in **E** was consistent with 4,25-dimethyltritriacontane. Peaks **E** and **F** located the first cyclopropyl group at C-3/C-4, in agreement with the NMR data. Peak **D** was a mixture of two internally branched isomers, which could not be separated by GC. The ion pairs *m*/*z* 210/211 and 309 as well as *m*/*z* 224/225 and 295 suggest that these compounds are 9,19- and 9,20-dimethyltritriacontane, localizing the second cyclopropane unit. Finally, peaks **B** and **C** (mass spectrum Figure 3b) are trimethyldotriacontanes, in agreement with the previous data. All mass spectra of peaks **B** and **D**–**G** are shown in the Supporting Information (Figures S1–S5). Taking these data together, there are two possible solutions for the position of the substituents in **A**. The methyl group can be located either between the two cyclopropane units (**7**) or outside them (**4**, Figure 4). The mass spectrum of peak **C**, consisting of two positional isomers originating from the internal cyclopropane, is formally consistent with the fragmentation of the hydrogenation products **5** and **6** or **8** and **9**, respectively (Figure 4). However, it is well known that an even-numbered ion formed by cleavage adjacent to the methyl group and H transfer is of higher intensity than the simple cleavage product ion when no other further branches occur within this fragment.^18,25^ Such an occurrence can be observed for ion *m*/*z* 140, which is of much higher intensity compared to *m*/*z* 141 in all spectra **B**–**G**. In the spectrum of **D**, this can also be observed for ions *m*/*z* 210/211 and 224/225. At the lower end of the mass spectra, this appearance is not visible due to the high overlap of the respective ions with ions produced by various other fragmentation pathways. We also performed hydrogenation with deuterium gas (Figure S7). During the ring-opening reactions, extensive D scrambling occurred around the original cyclopropane positions, obscuring all peaks. However, ions *m*/*z* 140/141 remained unchanged, indicating that the methyl group is far away from the cyclopropanes, further confirming its position at C-24 (Figure S7c and S7d).

In summary, these data suggest that compound **A** is structure **4**, 3,4,13,14-bismethylene-24-methyldotriacontane. To verify the structure, we carried out the synthesis of **4**.

### Total Synthesis

**Figure 5:**
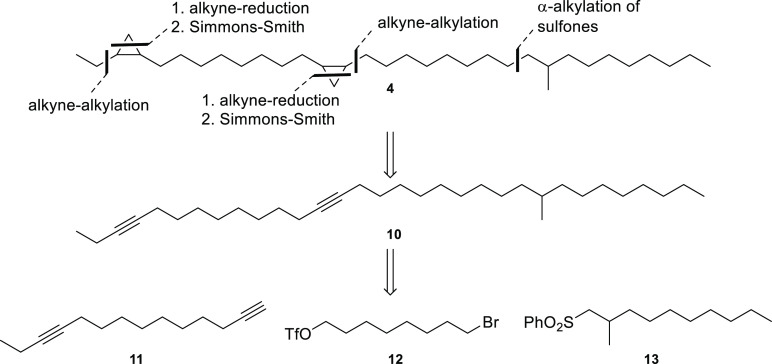
Retrosynthetic approach toward the synthesis of **4**.

Our retrosynthetic plan is shown in Figure 5. The key precursor is the dialkyne **10**. It allows a late-stage functionalization via Lindlar hydrogenation, yielding a *cis,cis*-alkadiene, or dissolving metal (Birch) reduction to access the *trans*,*trans*-alkadiene, if needed. The double-bond configuration can be used to establish the configuration of the cyclopropanes, which can be accessed via a modified Simmons–Smith reaction.^26^ The precursor **10** can be cleaved into three simple building blocks (**11**, **12**, **13**). Building blocks **11** and **13** contain nucleophilic carbons after activation, the terminal alkyne, and the α-carbon of the sulfone. These nucleophiles can be exploited to substitute the two distinguishable leaving groups of **12**.

**Scheme 1:**
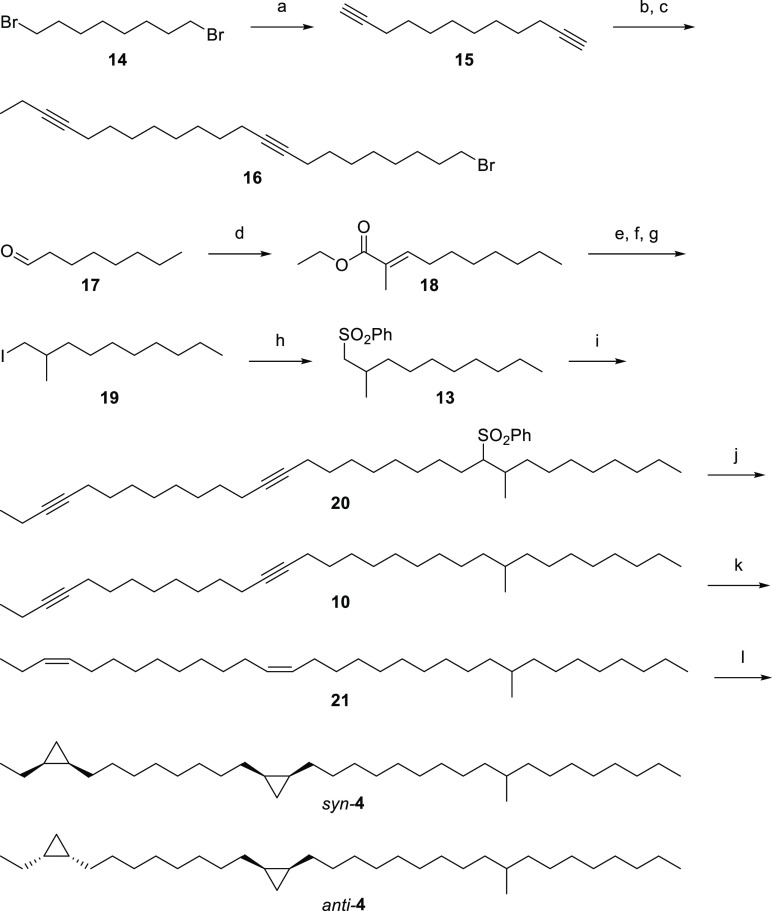
Synthesis of *rac*-Biscyclopropanes *syn-* and *anti-***4** (a) HCCLi, NaI, DMSO, 0–8 °C, 1.5 h, 77%; (b) *n*BuLi, then EtI, THF, 65 °C, 1 h, 57%; (c) *n*BuLi, then **7**, Et_2_O, from −78 °C to rt, 15 h, 59%; (d) Ph_3_P=C(CH_3_)CO_2_Et, CH_2_Cl_2_, rt, 17 h, *E*: 79%; (e) Pd/C, H_2_, MeOH, rt, 3 h, 99%; (f) LiAlH_4_, THF, rt, 16 h, quant.; (g) I_2_, PPh_3_, ImH, CH_2_Cl_2_, rt, 3.5 h, 76%; (h) PhSO_2_Na, DMF, rt, 62 h, 50 °C, 1 h, 77%; (i) *n*BuLi, LiI, −78 °C, 1 h then **16**, rt, 1 h, THF/HMPA (16:2), 42%; (j) Mg, MeOH, 65 °C, 5 h, 82%; (k) Lindlar-cat., quinoline, hexane, rt, 0.5 h, 85%; (l) AlEt_3_, CH_2_I_2_, CH_2_Cl_2_, rt, 17 h, 95%.

The building block **11** was prepared from 1,8-dibromooctane (**14**) (Scheme 1). First, both bromides were substituted with lithium acetylide to give the dialkyne **15**, which was then selectively alkylated with subequimolar amounts of ethyl iodide to form **11**. In the second alkylation step, **11** selectively substituted the triflate group of **12**, obtained from 8-bromooctan-1-ol. This temperature-controlled approach gave **16** with a good leaving group already installed.^27^ The third building block **13** was synthesized starting with a Wittig reaction from octanal (**17**). The resulting unsaturated ester **18** was converted into iodide **19** via Pd-catalyzed hydrogenation, LiAlH_4_ reduction of the ester, and iodination. Finally, substitution with sodium benzenesulfinate afforded the sulfone **13**.

The two building blocks **16** and **13** were then connected by α-alkylation of the sulfone. The sulfone group of the product **20** was cleaved under reducing conditions with Mg in MeOH to afford the key dialkyne **10**. Lindlar hydrogenation afforded the *cis,cis*-alkadiene **21**. All hydrocarbon products were routinely purified by argentation flash chromatography to allow removal of unwanted isomers. In the following Simmons–Smith-type cyclopropanation with triethylaluminum,^26^ the target compounds **4** were finally prepared. Each cyclopropane ring was selectively formed in a cis configuration,^26^ but both rings were formed independently. Therefore, a mixture of the syn (3R*,4S*,13S*,14R*-**4**) or anti (3R*,4S*,13R*,14S*-**4**) arrangement of the cyclopropane units toward each other was obtained. Each of these products consists of two diastereomers because of the stereogenic methyl group at C-24. In spite of extensive efforts, we were not able to separate the isomers, neither by preparative argentation flash chromatography nor by GC on polar, apolar, or chiral phases. This may be due to the isolated nature of the internal cyclopropanes and the lack of functional groups needed for better interaction with the stationary phase. In addition, NMR spectra showed only one set of signals, making it impossible to distinguish the diastereomers. For comparison, the group of Curran^28^ was able to assign very small shift differences between methyl groups separated by five CH_2_ groups in methyl-branched hydrocarbons. In **4**, the two cyclopropane groups and the methyl group are separated by eight or nine CH_2_ groups, making NMR discrimination between diastereomers impossible.

**Figure 6:**
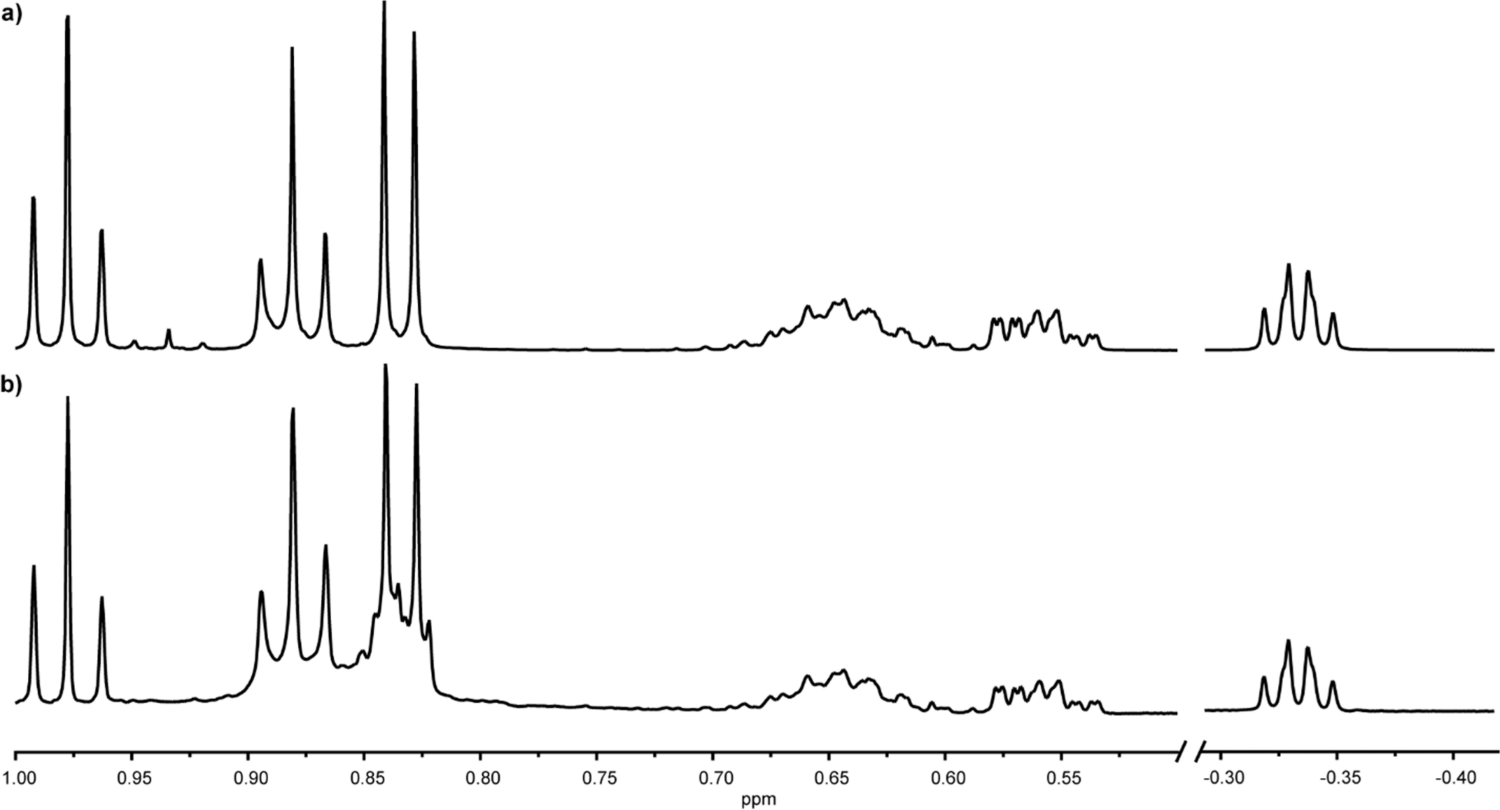
Direct comparison of the characteristic ^1^H NMR (500 MHz, CDCl_3_) shifts of (a) the synthetic compound **4** and (b) the natural product **A**.

The NMR data of the synthetic mixture of **4** and natural product **A** fit perfectly (Table 1, Figure 6), proving that the postulated carbon skeleton is correct, which is reinforced by identical mass spectra and gas chromatographic retention indices (*I*_syn_ 3446; *I*_nat_ 3446). We also performed a microhydrogenation with the synthetic **4** (Figure S6). The mass spectra were identical with those found in the hydrogenated sample of compound **A**. Figure S6b shows the mass spectrum of the major compound **K**, which is identical with the spectrum of **C** in Figure 3b. The reproducibility of the microhydrogenation further confirms the structure of **A**.

**Scheme 2:**
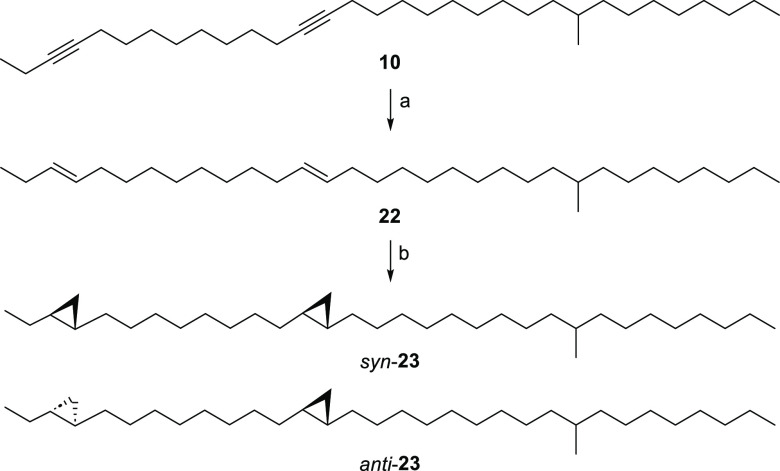
Synthesis of *trans*,*trans*-**23** by Birch Reduction (a) Na, NH_3_(l), HMPA, −33 °C, 2%; Lindlar-cat., quinoline, hexane, rt, 0.5 h, 2%; (b) AlEt_3_, CH_2_I_2_, CH_2_Cl_2_, rt, 17 h.

Although the structure elucidation so far seemed convincing, we could not completely rule out that *trans*-cyclopropanes would have the same gas chromatographic retention index as the cis compounds. Therefore, a small amount of **10** was subjected to Birch reduction^29^ to yield the *trans*,*trans*-alkadiene **22** (Scheme 2). Due to the formation of byproducts (*cis,cis*- and *cis,trans*-alkadiene as well as alkenynes) and the difficulty of separation, only a small amount of pure **22** was isolated. The subsequent cyclopropanation afforded the *trans,trans*-cyclopropanes **23**. The retention index of **23** (*I* 3379) is considerably smaller compared to that of **4** (*I* 3446), although the dotriacontadiene precursor **22** did not show any difference in *I* (**21**, *I* 3210; **22**, *I* 3209). Each syn-configured cyclopropane in **4** increases *I* by 118 compared to its synthetic precursor **21**, while *anti*-cyclopropanes lead only to an increase of 85. These results further support **4** to be the structure of **A**.

### Mass Spectrometry and Gas Chromatography of Long-Chain Cyclopropane Hydrocarbons

**Figure 7:**
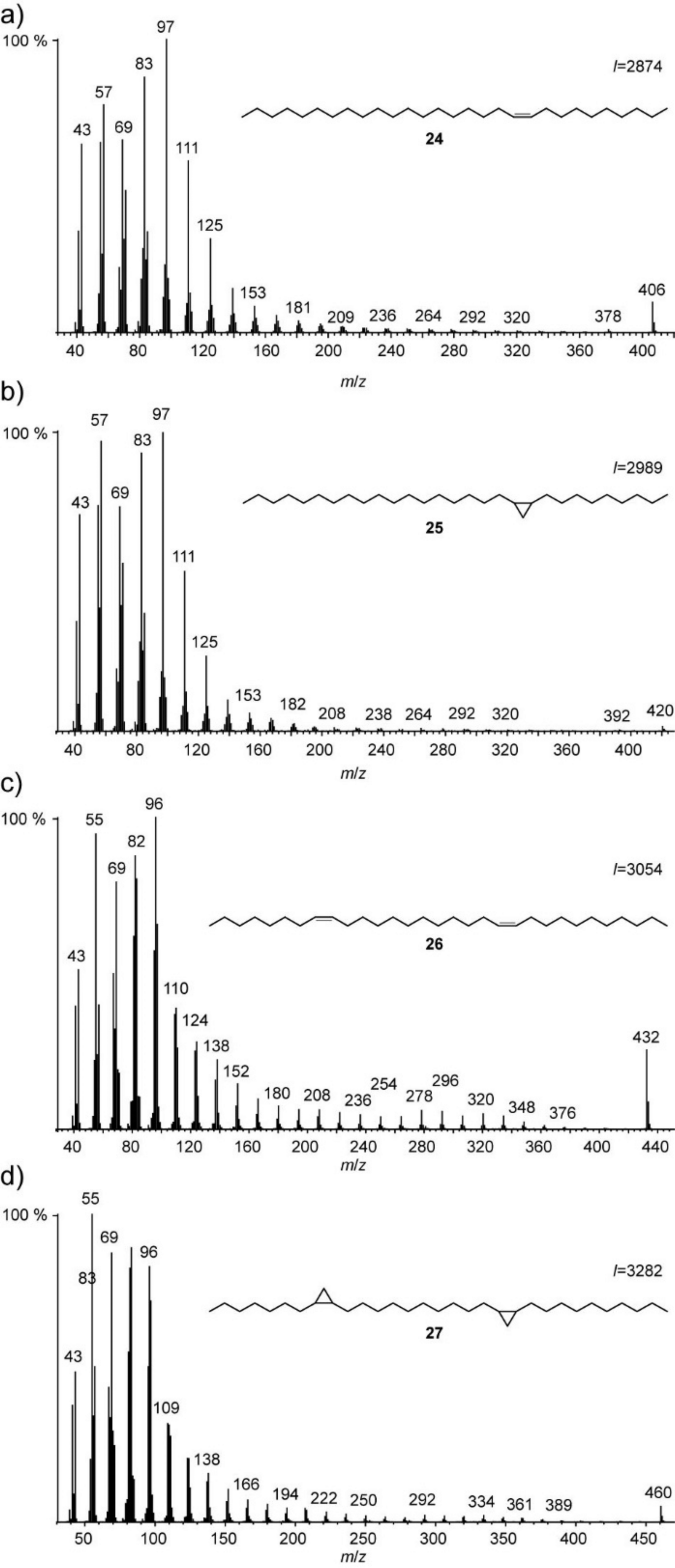
Comparison of the mass spectra of (a) (*Z*)-nonacos-10-ene (**24**), (b) *cis*-10,11-methylenenonacosane (**25**), (c) (8*Z*,20*Z*)-hentriaconta-8,20-diene (**26**), and (d) *cis,cis*-8,9,20,21-bismethylenehentriacontane (**27**).

As mentioned, long-chain alkenes are commonly found in complex mixtures of insect cuticular hydrocarbons.^10^ Because of the striking similarities in the mass spectra of **4** and **23** and their synthetic precursors **21** and **22**, we synthesized mono- and dicyclopropanes from various alkenes available to us. The products allowed us to investigate the mass spectra and gas chromatographic retention indices of these compounds.

(*Z*)-Nonacos-10-ene (**24**) and (8*Z*,20*Z*)-hentriaconta-8,20-diene^30^ (**26**) were converted into the corresponding cyclopropanes as described above. The mass spectra of the alkenes and the corresponding cyclopropanes (**25** and **27**) are shown in Figure 7. Except for the higher molecular ion and a slightly increased intensity of ions in the higher mass range in the alkenes, the spectra of both compound types are very similar. It seems doubtful that these differences are significant enough to differentiate between the compounds when analyzing complex mixtures.

**Figure 8:**
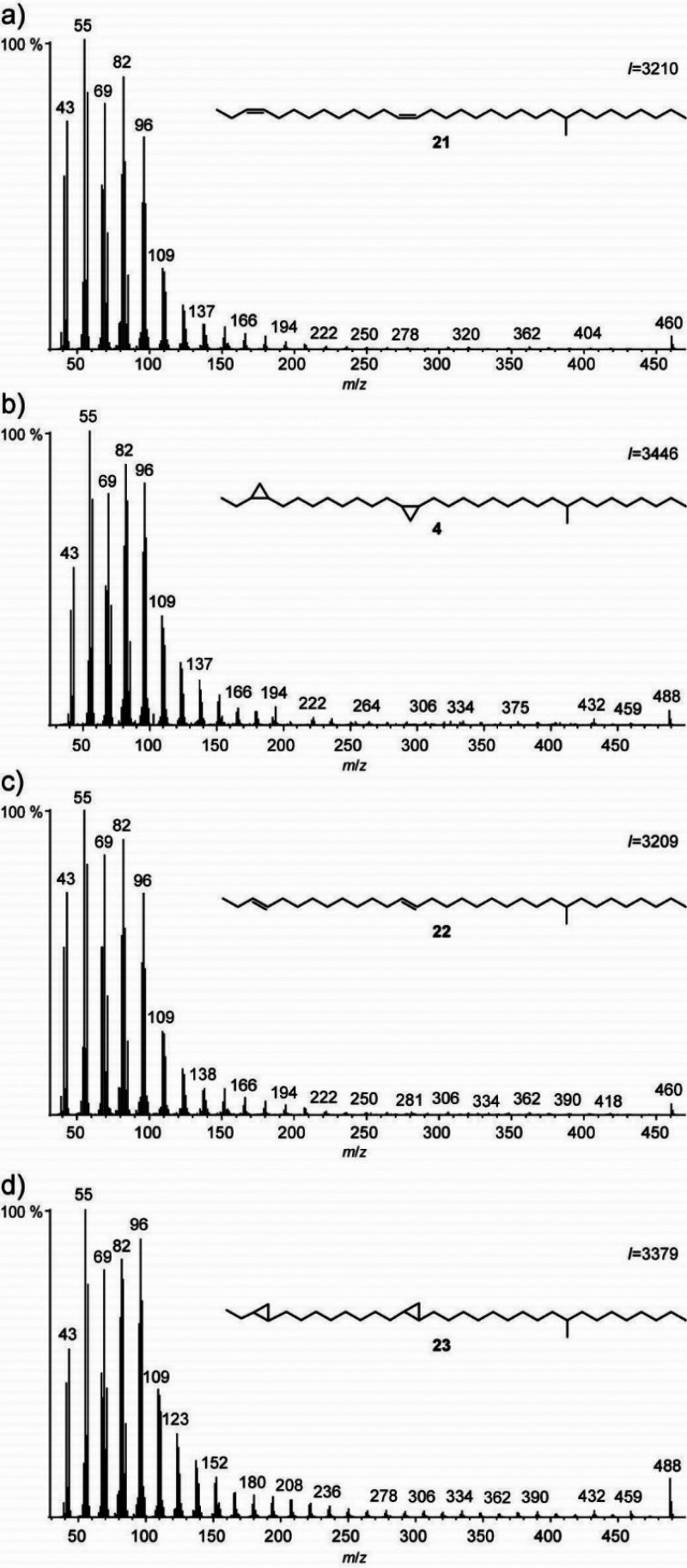
Comparison of the mass spectra of a) (3*Z*,13*Z*)-24-methyldotriaconta-3,13-diene (**21**), (b) *cis,cis*-3,4,13,14-bismethylene-24-methyldotriacontane (**4**), (c) (3*E*,13*E*)-24-methyldotriaconta-3,13-diene (**22**), and (d) *trans,trans*-3,4,13,14-bismethylene-24-methyldotriacontane (**23**).

By comparison of the spectra of the synthetic *cis-* and *trans*-alkadienes **21** and **22** and the *cis-* and *trans-*cyclopropanes **4** and **23**, no reliable differences between the spectra can be observed (Figure 8). Even more so, the intensity of the molecular ions is now independent of the compound structure, being most intense in trans-configured cyclopropanes.

The retention index *I* of compounds **4**, **25**, and **27** increases by about 115 per added *syn*-cyclopropane, making it difficult to distinguish them from the value of *I* of a CH_2_-elongated alkene, which should show an increase of 100. For example, complex mixtures of triacontenes eluting in the range *I* 2970–2989 and hentriacontadienes (*I* 3245–3270) have been reported from the sugar cane borer, *Diatraea saccharalis*.^31^ The values of **25** and **27** fall into the range of the alkenes. This underscores the importance of not relying on *I* and mass spectra alone when analyzing arthropod alkenes. Dimethyldisulfide addition is commonly used for the localization of double-bond positions in linear alkenes,^21^ but the absence of reaction may not be due to the presence of cyclopropanes but to experimental failure. In contrast, hydrogenation with Pd/C yields linear alkanes. In the case of cyclopropanes, a mixture of methyl-branched alkanes is formed, while alkenes would give one alkane, allowing a positive decision on the correct structure.

Natural cyclopropanes are biosynthesized by various pathways in microorganisms,^32^ often occurring in complex terpenes or polyketides such as shizukaol A^33^ or jawsamycin^34^ or in acids such as mycolic acids of *Mycobacterium tuberculosis,*(35) but their occurrence in arthropods is rare. Females of the millipede *Graphidostreptus tumuliporus* (Myriapoda: Diplopoda) produce large amounts of *cis*-9,10-methyleneoctadecanoic acid and related compounds.^36^*cis*-Cyclopropanes are usually formed by methylation of a double bond with *S*-adenosylmethionine forming a secondary cation, followed by ring closure and H abstraction from the methyl group.^37^ In the few cases investigated so far, springtail cuticular hydrocarbons lack the complexity of the multicomponent insect hydrocarbon layer^3,7,12,13^ using one or a few compounds, one of which is often cholesterol. This is also the case for sarekensane (**4**), the primary function of which might be desiccation prevention, as is the case with other arthropod cuticular hydrocarbons.^38^ However, its unique structure may also indicate a signaling function, perhaps ensuring the aggregation of the springtails. The finely tuned length of the hydrocarbon chain and the position of the cyclopropane rings may also lead to specific properties. In cyclopropane fatty acids, the cyclopropane group has been shown to enhance lateral diffusion while stabilizing the lipid layer compared to unsaturated acids.^39^ Similar effects may also be operative in the *V. sarekensis* alkane. This might be an adaptation to the harsh conditions in the high alpine regions in which *V. sarekensis* lives.

## Conclusion

Cyclopropane hydrocarbons are a new structural variant of arthropod cuticular hydrocarbons. They have not been reported before from nature and add to the large structural variability of springtail epicuticular hydrocarbons. The structural analysis of these compounds is difficult because the mass spectra and gas chromatographic retention indices are similar to those of common alkenes and requires the combination of different methods, including GC/MS and microderivatization. The specificity of the cyclopropane localization may hint to a specialized function of this unique compound.

## Experimental Section

### General Experimental Procedures

IR spectra were measured on a Bruker Tensor 27 (diamond-ATR) or Agilent Technologies 7890B gas chromatograph equipped with a HP5 phase connected to a Dani Instruments DiscovIR DDFTIRInterface. NMR spectra were recorded either on an Avance III HD 300N (^1^H NMR, 300 MHz; ^13^C NMR, 76 MHz), AVII 400 (^1^H NMR, 400 MHz; ^13^C NMR, 101 MHz), or AVIIIHD-500 MHz (^1^H NMR, 500 MHz; ^13^C NMR, 125 MHz) instrument. Mass spectra were recorded with a combination of an Agilent Technologies 5977B gas chromatograph connected to an Agilent Technologies 8860 Series MSD. Gas chromatographic retention indices were calculated against a series of *n*-alkanes according to van den Dool and Kratz^40^ using a standard HP-5 phase. Column chromatography: silica 60 (0.063–0.200 mm, 70–230 mesh ASTM). Thin layer chromatography (TLC): Polygram SIL G/UV silica 60, 0.20 mm. Compounds were stained with potassium permanganate solution. All reactions were performed in oven-dried glassware under a nitrogen atmosphere. Solvents were dried according to standard procedures.

### Biological Material and Isolation

*V. sarekensis* was collected at an altitude of 1800 m in the Jotunheimen mountains in southern Norway. They were sampled from a mass occurrence of tens of thousands of animals aggregating on the surface of a pond. The springtails were collected in jars covered with 10% activated charcoal in plaster of Paris and transported to the laboratory in Oslo. There the springtails (∼5 g) were extracted for 15 min with pentane (Ultrasolv, Merck) in batches in 5 mL vials. The springtails were separated, and the extracts were sent to Braunschweig. The extracts were combined, and the concentrated extract was applied to a short silica column made from a Pasteur pipet. Fractions were obtained by elution with pentane. The sarekensane-containing fractions were combined, the solvent was removed, and the purified compound was subjected to NMR analysis.

### Microderivatization

#### Hydrogenation

A 100 μL amount of a solution in pentane with a concentration suitable for GC analysis was placed in a 1.5 mL vial equipped with a 200 μL insert. A minute amount of Pd/C was added. A hydrogen atmosphere was applied for 3 h or until GC/MS analysis showed complete conversion. The catalyst was then removed by filtration through Celite pad.

#### Cyclopropanation

A 10 μL amount of a solution of an alkene in CH_2_Cl_2_ with a concentration suitable for GC analysis was placed in a 1.5 mL vial fitted with a 200 μL insert. Diiodomethane (3 μL) and AlEt_3_ (1 M in hexane, 10 μL) were added, and the resulting mixture was left at room temperature (rt) for 16 h. Then, NaF (20 mg) and H_2_O (200 μL) were added. After 10 min, the resulting jelly was extracted with CH_2_Cl_2_ (2 × 100 μL). The organic extracts were combined, filtered over MgSO_4_ in a Pasteur pipet, and analyzed by GC/MS.

### Synthesis

#### 8-Bromooctyl Trifluoromethanesulfonate

Triflic acid anhydride (2.428 g, 8.607 mmol, 1.2 equiv) and pyridine (579 μL, 7.173 mmol, 1.0 equiv) were added to a solution of 8-bromo-1-octanol (1.500 g, 7.713 mmol, 1.0 equiv) in CH_2_Cl_2_ (28 mL) cooled to −15 °C. The resulting mixture was stirred for 1 h at 0 °C, diluted with hexane (60 mL), and passed through a Celite pad.^27^ The solvent was removed under reduced pressure to give 8-bromooctyl trifluoromethanesulfonate as a light-brown liquid (2.302 g, 6.747 mmol, 94%), which was used without further purification in the next step. IR (neat) ν_max_ 2933, 2859, 1409, 1243, 1199, 1143, 1028, 923, 828, 612, 573; ^1^H NMR (CDCl_3_, 300 MHz) δ 4.58 (t, *J* = 6.5 Hz, 2H), 3.45 (t, *J* = 6.8 Hz, 2H), 1.95–1.82 (m, 4H), 1.54–1.35 (m, 8H); ^13^C NMR (CDCl_3_, 75 MHz) δ 77.6 (CH_2_), 33.8 (CH_2_), 32.6 (CH_2_), 29.2 (CH_2_), 28.7 (CH_2_), 28.5 (CH_2_), 27.9 (CH_2_), 24.97 (CH_2_).

#### Dodeca-1,11-diyne (**15**)

A solution of **14** (3.850 g, 14.153 mmol, 1.0 equiv) in DMSO (7 mL) was added over 15 min to a cooled suspension of lithium acetylide (3.258 g, 35.383 mmol, 2.5 equiv) in DMSO (29 mL). After complete addition, the mixture was stirred for 1.5 h at 10–15 °C and diluted with Et_2_O (150 mL).^41^ The resulting mixture was washed with H_2_O (3 × 100 mL) and brine (100 mL). After drying over MgSO_4_, the solvent was removed under reduced pressure. The resulting residue was purified by flash chromatography (pentane) to give **15** as a colorless oil (1.756 g, 10.819 mmol, 77%). IR (neat) ν_max_ 3300, 2930, 2856, 1461, 1435, 722, 625 cm^–1^; ^1^H NMR (CDCl_3_, 300 MHz) δ 2.26–2.06 (4H, dt, *J* = 7.04 Hz, *J* = 2.63 Hz), 1.94 (2H, t, *J* = 2.65 Hz), 1.60–1.24 (12H, m); ^13^C NMR, DEPT (CDCl_3_, 75 MHz) δ 84.7 (C_q_), 68.1 (CH), 28.9 (CH_2_), 28.7 (CH_2_), 28.4 (CH_2_), 18.4 (CH_2_); EIMS *m*/*z* 162 (<1), 133 (2), 119 (12), 93 (34), 91 (51), 81 (57), 80 (25), 79 (100), 67 (65), 55 (45), 53 (36), 41 (71), 39 (49); HRCIPMS *m*/*z* 161.13237 [M – H]^+^ (calcd for C_12_H_17_, 161.13248).

#### Tetradeca-1,11-diyne (**11**)

*n-*Butyl lithium (1.9 M in hexane, 3.160 mL, 5.995 mmol, 1.1 equiv) was added to a stirred solution of **15** (1.769 g, 10.900 mmol, 2.0 equiv) in THF (50 mL) at −78 °C. After 5 min at −78 °C, the reaction mixture was allowed to warm to rt and stirred for 20 min. The mixture was heated to 60 °C, and ethyl iodide (438 μL, 5.450 mmol, 1.0 equiv) was added over 20 min. After heating at reflux for 1.5 h, the reaction was cooled to rt and quenched by addition of sat. NH_4_Cl solution (50 mL).^42^ The mixture was extracted with pentane (3 × 50 mL); the combined organic phases were dried over MgSO_4_ and concentrated under reduced pressure. The residue was purified by flash chromatography (pentane) to give **11** as a colorless oil (589 mg, 3.096 mmol, 57%) and unreacted starting material (1.178 g, 7.259 mmol). IR (neat) ν_max_ 3305, 2973, 2929, 2855, 1741, 1459, 1437, 1370, 1324, 1225, 723, 626 cm^–1^; ^1^H NMR (CDCl_3_, 300 MHz) δ 2.22–2.09 (6H, m), 1.94 (1H t, *J* = 2.65 Hz), 1.58–1.26 (12H, m), 1.11 (3H, t, *J* = 7.38 Hz); ^13^C NMR, DEPT (CDCl_3_, 75 MHz) δ 84.8 (C_q_), 81.6 (C_q_), 79.5 (C_q_), 68.1 (CH), 29.1 (CH_2_), 29.0 (CH_2_), 29.0 (CH_2_), 28.8 (CH_2_), 28.7 (CH_2_), 28.5 (CH_2_), 18.7 (CH_2_), 18.4 (CH_2_), 14.4 (CH_3_), 12.4 (CH_2_); EIMS *m*/*z* 191 (<1), 175 (1), 161 (6), 107 (31), 95 (50), 93 (45), 91 (32), 81 (67), 79 (71), 69 (28), 67 (100), 55 (68), 41 (60); HREIMS *m*/*z* 189.16377 [M – H]^+^ (calcd for C_14_H_21_, 189.16378).

#### 22-Bromodocosa-3,13-diyne (**16**)

A solution of **11** (728 mg, 3.825 mmol, 1.0 equiv) in THF (8 mL) was degassed by the freeze–pump–thaw technique (three cycles). The solution was cooled to −78 °C, *n*BuLi (2.21 mL, 4.207 mmol, 1.1 equiv) was added, and the resulting mixture was allowed to slowly warm in the cooling bath. After 30 min, 8-bromooctyl trifluoromethanesulfonate (1.957 g, 5.737 mmol, 1.5 equiv) in THF (5 mL) was added dropwise at −78 °C.^42^ The reaction mixture was allowed to warm to rt, after 1.5 h. H_2_O was added (50 mL) and finally extracted with pentane (3 × 50 mL). The combined organic phases were dried over MgSO_4_ and concentrated under reduced pressure. The residue was purified by flash chromatography (pentane/Et_2_O; 100:1). This gave a mixture of unreacted **11** and the desired diyne **16**, which was then taken up in a small amount of CH_2_Cl_2_ and added to a stirred solution of AgNO_3_ in MeOH (30 mL).^43^ After 10 min of stirring, the white precipitate, the Ag acetylide of **11**, was filtered off. H_2_O (30 mL) was added to the filtrate, and the resulting mixture was extracted with pentane (3 × 30 mL), dried over MgSO_4_, and concentrated under reduced pressure to give **16** as a colorless oil (626 mg, 1.642 mmol, 43%). IR (neat) ν_max_ 2927, 2854, 1460, 1436, 1326, 1248, 1215, 723, 646, 557, 534 cm^–1^; ^1^H NMR (CDCl_3_, 300 MHz) δ 3.41 (1H, t, *J* = 6.9 Hz), 2.22–2.08 (3H, m), 1.86 (1H, q, *J* = 7.1 Hz), 1.54–1.22 (9H, m), 1.12 (1H, t, *J* = 7.4 Hz); ^13^C NMR, DEPT (CDCl_3_, 75 MHz) δ 81.7 (C_q_), 80.4 (C_q_), 80.3 (C_q_), 79.7 (C_q_), 34.2 (CH_2_), 33.0 (CH_2_), 29.3 (CH_2_), 29.2 (CH_2_), 29.2 (CH_2_), 29.1 (CH_2_), 29.0 (CH_2_), 28.9 (CH_2_), 28.8 (CH_2_), 28.3 (CH_2_), 18.9 (CH_2_), 14.5 (CH_3_), 12.6 (CH_2_); EIMS *m*/*z* 353 (1), 189 (18), 107 (32), 95 (50), 93 (45), 91 (33), 81 (69), 79 (68), 69 (29), 67 (100), 55 (70), 41 (61); HRCIPMS *m*/*z* 381.19763 [M(^81^Br) – H]^+^ (calcd for C_22_H_36_^81^Br, 381.28758).

#### Ethyl (*E*)-2-Methyldec-2-enoate (**18**)

Octanal (**17**, 1.557 g, 12.141 mmol, 1.1 equiv) was added to a solution of (carbethoxyethylidene)triphenylphosphorane (4.000 g, 11.038 mmol, 1.0 equiv) in CH_2_Cl_2_ (6 mL) cooled to 0 °C.^44^ The mixture was stirred at rt for 17 h. The solvent was removed under reduced pressure. Et_2_O was added, and the formed white precipitate was removed by filtration over a silica pad. The filtrate was concentrated and purified by flash chromatography (pentane/Et_2_O; 40:1) to obtain **18** as a colorless oil (1.846 g, 8.694 mmol, 79%). IR (neat) ν_max_ 2956, 2925, 2856, 1710, 1650, 1463, 1387,1367, 1265, 1208, 1177, 1139, 1097, 136, 870, 743, 667, 658, 568, 542 cm^–1^; ^1^H NMR (300 MHz, CDCl_3_) δ 6.76 (1H, td, *J* = 7.5, 1.3 Hz), 4.19 (2H, q, *J* = 7.1 Hz), 2.16 (2H, q, *J* = 7.2 Hz), 1.83 (3H, s), 1.51–1.37 (2H, m), 1.37–1.18 (11H, m), 0.88 (3H, t, *J* = 6.7 Hz); ^13^C NMR, DEPT (CDCl_3_, 75 MHz) δ 168.5 (C), 142.6 (CH), 127.8 (C), 60.5 (CH_2_), 31.9 (CH_2_), 29.5 (CH_2_), 29.3 (CH_2_), 28.8 (CH_2_), 28.7 (CH_2_), 22.8 (CH_2_), 14.4 (CH_3_), 14.2 (CH_3_), 12.5 (CH_3_). EIMS *m*/*z* 212 (11, [M]^+^), 167 (40), 115 (78), 113 (35), 102 (100), 87 (65), 69 (54), 67 (35), 55 (73), 43 (55), 41 (60); HRCIPMS *m*/*z* 213.18472 [M + H]^+^ (calcd for C_13_H_25_O_2_, 213.18491).

#### Ethyl 2-Methyldecanoate

A suspension of **18 (**500 mg, 2.354 mmol) and Pd/C (118 mg) in MeOH was stirred for 1 h under a H_2_ atmosphere. The mixture was filtered through a Celite pad, and the solvent was removed under reduced pressure.^45^ Ethyl 2-methyldecanoate was obtained as a colorless oil (501 mg, 2.336 mmol, 99%) and used without further purification. ^1^H NMR (CDCl_3_, 300 MHz) δ 4.13 (2H, q, *J* = 7.2 Hz), 2.41 (1H, dq, *J* = 13.9, 7.0 Hz), 1.73–1.55 (1H, m), 1.51–1.20 (16H, m), 1.13 (3 H, d, *J* = 7.0 Hz), 0.88 (3H, t, *J* = 6.9 Hz); ^13^C NMR (CDCl_3_, 75 MHz) δ 177.1 (C), 60.2 (CH_2_), 39.7 (CH), 34.0 (CH_2_), 32.0 (CH_2_), 29.7 (CH_2_), 29.6 (CH_2_), 29.4 (CH_2_), 27.4 (CH_2_), 22.8 (CH_2_), 17.2 (CH_2_), 14.4 (CH_3_), 14.2 (CH_3_); HREIMS *m*/*z* 215.20056 [M + H]^+^ (calcd for C_13_H_27_O_2_, 215.20056).

#### 2-Methyldecan-1-ol

Lithium aluminum hydride (117 mg, 3.085 mmol, 1.1 equiv) was added to a solution of ethyl 2-methyldecanoate (601 mg, 2.805 mmol, 1.0 equiv) in THF (3 mL) cooled to 0 °C. After stirring 0.5 h at 0 °C, the mixture was allowed to warm to rt and was stirred for another 0.5 h. Saturated Rochelle solution (5 mL) was added. The mixture was stirred for 1 h. The phases were separated, and the aqueous phase was extracted with Et_2_O (3 × 5 mL).^46^ The combined organic phases were washed with brine (5 mL) and dried over MgSO_4_. The solvent was removed under reduced pressure to give 2-methyldecan-1-ol as a colorless oil (483 mg, 2.803 mmol, quant.), which was used directly in the next step. IR (neat) ν_max_ 3314, 2956, 2922, 2854, 1462, 1377, 1037, 939, 721, 680, 654, 647, 620, 594, 554, 540 cm^–1^; ^1^H NMR (CDCl_3_, 300 MHz) δ 3.46 (2H, ddd, *J* = 29.6, 10.5, 6.2 Hz), 1.73–1.51 (1H, m), 1.47–1.17 (14H, m), 1.17–1.03 (1H, m), 0.96–0.83 (6H, m); ^13^C NMR, DEPT (CDCl_3_, 75 MHz) δ 68.6 (CH_2_), 35.9 (CH), 33.3 (CH_2_), 32.0 (CH_2_), 30.1 (CH_2_), 29.8 (CH_2_), 29.5 (CH_2_), 27.1 (CH_2_), 22.8 (CH_2_), 16.7 (CH_3_), 14.3 (CH_3_). EI-MS *m*/*z* 154 (2), 85 (37), 83 (32), 71 (42), 70 (44), 57 (100), 56 (61), 55 (57), 69 (52), 43 (84), 41 (72); HRCIPMS *m*/*z* 171.17415 [M – H]^+^ (calcd for C_11_H_23_O, 171.29959).

#### 1-Iodo-2-methyldecane (**19**)

Iodine (2.525 g, 9.947 mmol, 2.0 equiv) was added to a solution of triphenylphosphane (2.608 g, 9.947 mmol, 2.0 equiv) and imidazole (677 mg, 9.947 mmol, 2.0 equiv) in CH_2_Cl_2_ (20 mL). The resulting mixture was stirred for 30 min at rt. Then, 2-methyldecan-1-ol **(**857 mg, 4.974 mmol, 1.0 equiv) was added at 0 °C. The mixture was stirred for 3.5 h, and sat. Na_2_S_2_O_3_ solution (20 mL) was added, followed by CH_2_Cl_2_ (50 mL) and sat. NaHCO_3_ solution (50 mL). After phase separation, the aqueous phase was extracted with CH_2_Cl_2_ (2 × 50 mL).^47^ The combined organic phases were dried over MgSO_4_, and the solvent was removed under reduced pressure. The resulting residue was purified by flash chromatography (pentane) to give **19** as a colorless oil (1402 mg, 4.970 mmol, quant.). IR (neat) ν_max_ 2956, 2922, 2852, 1460, 1376, 1324, 1193, 722, 605, 590, 533 cm^–1^; ^1^H NMR (CDCl_3_, 300 MHz,) δ 3.19 (2H, ddd, *J* = 15.5, 9.5, 5.3 Hz), 1.55–1.12 (15H, m), 0.97 (3H, d, *J* = 6.5 Hz), 0.88 (3H, t, *J* = 6.8 Hz); ^13^C NMR, DEPT (CDCl_3_, 75 MHz) δ 36.6 (CH), 34.9 (CH_2_), 32.0 (CH_2_), 29.8 (CH_2_), 29.7 (CH_2_), 29.4 (CH_2_), 27.1 (CH_2_), 22.8 (CH_2_), 20.8 (CH_2_), 18.1 (CH_2_), 14.3 (CH_2_); EIMS *m*/*z* 282 (<1, [M]^+^), 155 (11), 85 (35), 71 (49), 69 (10), 57 (100), 56 (11), 55 (25), 43 (66), 41 (45), 42 (11); HRCIPMS *m*/*z* 281.07601 [M – H]^+^ (calcd for C_11_H_22_I, 281.07607).

#### ((2-Methyldecyl)sulfonyl)benzene (**13**)

Sodium benzenesulfinate (1.396 g, 8.504 mmol, 1.5 equiv) was added to a stirred solution of **19** (1.600 g, 5.670 mmol, 1.0 equiv) in DMF (10 mL). The resulting mixture was stirred for 62 h at rt. H_2_O (20 mL) was then added, and the mixture was extracted with Et_2_O (3 × 20 mL). The combined organic phases were washed with sat. NaHCO_3_ solution (20 mL) and brine (20 mL). After drying over MgSO_4_, the solvent was removed under reduced pressure.^48^ The resulting residue was purified by flash chromatography (pentane/Et_2_O; 10:1) to give **13** as a colorless oil (1.286 g, 4.338 mmol, 77%). IR (neat) ν_max_ 2956, 2923, 2853, 1463, 1448, 1404, 1380, 1304, 1145, 186, 1024, 999, 830, 780, 741, 719, 689, 597, 570, 539 cm^–1^; ^1^H NMR (CDCl_3_, 300 MHz) δ 7.95–7.89 (2H, m), 7.69–7.62 (1H, m), 7.61–7.53 (2H, m), 3.08 (1H, dd, *J* = 14.1, 4.6 Hz), 2.92 (1H, dd, *J* = 14.1, 7.8 Hz), 2.05 (1H, dt, *J* = 14.5, 6.8 Hz), 1.47–1.12 (14H, m), 1.06 (3H, d, *J* = 6.7 Hz), 0.88 (3H, t, *J* = 6.8 Hz); ^13^C NMR, DEPT (CDCl_3_, 75 MHz) δ 140.3 (C), 133.6 (CH_Ar_), 129.4 (CH_Ar_), 128.0 (CH_Ar_), 62.7 (CH_2_), 36.9 (CH_2_), 32.0 (CH_2_), 29.6 (CH_2_), 29.6 (CH_2_), 29.4 (CH_2_), 28.7 (CH), 26.5 (CH_2_), 22.8 (CH_2_), 20. (CH_3_), 14.2 (CH_3_); EIMS *m*/*z* 296 ([M]^+^, < 1), 143 (100), 85 (30), 78 (33), 77 (58), 71 (42), 69 (28), 57 (81), 55 (47), 43 (63), 41 (56); HREIMS *m*/*z* 297.18835 [M + H]^+^ (calcd for C_17_H_29_O_2_S, 297. 18828).

#### ((9-Methyldotriaconta-19,29-diyn-10-yl)sulfonyl)benzene (**20**)

A solution of **13** (385 mg, 1.299 mmol, 1.0 equiv), LiI (2.226 g, 1.688 mmol, 1.3 equiv), and hexamethylphosphoramide (HMPA, 1.3 mL) in THF (9.9 mL) was degassed by the freeze–pump–thaw technique (three cycles). *n*-Butyl lithium (1.6 M in hexane, 890 μL) was added to the solution at −78 °C, and the reaction mixture was stirred at −78 °C for 1 h. Then, bromide **16** (594 mg, 1.558 mmol, 1.2 equiv) in THF (4 mL) was added, and the mixture was allowed to reach rt and stirred for a further 2 h. After acidification with HCl (1 M, 20 mL), the resulting mixture was extracted with Et_2_O (3 × 20 mL).^49^ The combined organic phases were washed with HCl (1 M, 2 × 20 mL) and brine (20 mL), dried over MgSO_4_, and concentrated under reduced pressure. The residue was purified by flash chromatography (pentane/Et_2_O; 10:1) to give **20** as a colorless oil (329 mg, 0.551 mmol, 42%). IR (neat) ν_max_ 2924, 2853, 1462, 1448, 1375, 1303, 1145, 1084, 1027, 723, 692, 676 612, 573, 547 cm^–1^; ^1^H NMR (CDCl_3_, 300 MHz) δ 7.93–7.84 (2H, m), 7.64 (1H, t, *J* = 7.3 Hz), 7.56 (2H, t, *J* = 7.3 Hz), 2.97–2.84 (1H, m), 2.23–1.97 (8H, m), 1.97–1.74 (1H, m), 1.74–1.54 (2H, m), 1.54–1.06 (41H, m), 1.06–0.94 (3H, m), 0.94–0.82 (3H, m); ^13^C NMR, DEPT (CDCl_3_, 75 MHz) δ 139.5 (C_q_), 133.5 (CH), 129.2 (CH), 128.7 (CH), 81.7 (C≡C), 80.4 (C≡C), 80.3 (C≡C), 79.7 (C≡C), 68.1 (CH), 36.0 (CH_2_), 32.0 (CH_2_), 31.8 (CH), 29.7 (CH_2_), 29.6 (CH_2_), 29.4 (CH_2_), 29.4 (CH_2_), 29.3 (CH_2_), 29.3 (CH_2_), 29.2 (CH_2_), 29.1 (CH_2_), 29.0 (CH_2_), 28.9 (CH_2_), 27.4 (CH_2_), 23.7 (CH_2_), 22.8 (CH_2_), 18.9 (CH_2_), 15.0 (CH_3_), 14.5 (CH_3_), 14.2 (CH_3_), 12.6 (CH_2_). HRESIMS *m*/*z* 619.45196 [M + Na]^+^ (calcd for C_39_H_64_O_2_SNa, 619.45192).

#### 24-Methyldotriaconta-3,13-diyne (**10**)

Magnesium turnings (326 mg, 13.400 mmol, 20 equiv) were added to a stirred solution of **20** (400 mg, 0.670 mmol, 1.0 equiv) in MeOH (20 mL). The resulting mixture was heated to reflux for 5 h. After cooling to rt, HCl (1 M, 60 mL) was carefully added. The mixture was extracted with pentane (3 × 20 mL). The combined organic phases were dried over MgSO_4_ and concentrated under reduced pressure.^50^ The residue was purified by argentation flash chromatography (SiO_2_·AgNO_3_; pentane) to give **10** as a colorless oil (252 mg, 0.552 mmol, 82%). IR (neat) ν_max_ 2922, 22853, 1739, 1461, 1373, 1327, 722, 583, 560, 548 cm^–1^; ^1^H NMR (CDCl_3_, 300 MHz) δ 2.22–2.09 (8H, m), 1.53–1.17 (44H, m), 1.17–1.00 (5H, m), 0.88 (3H, d, *J* = 6.7 Hz), 0.83 (3H, d, *J* = 6.4 Hz); ^13^C NMR, DEPT (CDCl_3_, 75 MHz) δ 81.6 (C), 803 (C), 80.2 (C), 79.6 (C), 37.1 (CH_2_), 32.8 (CH), 32.0 (CH_2_), 30.1 (CH_2_), 30.0 (CH_2_), 29.7 (CH_2_), 29.6 (CH_2_), 29.4 (CH_2_), 29.2 (CH_2_), 29.2 (CH_2_), 29.2 (CH_2_), 29.1 (CH_2_), 29.1 (CH_2_), 28.9 (CH_2_), 28.8 (CH_2_), 27.1 (CH_2_), 22.7 (CH_2_), 19.7 (CH_3_), 18.7 (CH_2_), 18.7 (CH_2_), 14.4 (CH_3_), 14.1 (CH_3_), 12.4 (CH_2_); EI MS *m*/*z* 456 (<1), 427 (8), 189 (51), 95 (68), 81 (82), 79 (57), 69 (48), 67 (100), 57 (76), 55 (79), 43 (85), 41 (62); HRCIPMS *m*/*z* 455.46136 [M – H]^+^ (calcd for C_33_H_59_ 455.46113).

#### (3*Z*,13*Z*)-24-Methyldotriaconta-3,13-diene (**21**)

A solution of quinoline (2 μL, 0.014 mmol, 0.1 equiv) in hexane (2 mL) was degassed by the freeze–pump–thaw technique. The Lindlar cat. (1.6 mg) was added, and the resulting mixture was stirred 20 min at rt. Diyne **10** (52 mg, 0.114 mmol, 1.0 equiv) was added, and the mixture was stirred for 1 h under a H_2_ atmosphere.^51^ As no conversion was observed, additional Lindlar cat. (4.0 mg) was added. Conversion was complete after a further 30 min under H_2_ atmosphere. The catalyst was filtered off through a Celite pad with a mixture of pentane/Et_2_O (1:1, 6 mL), and the solution was concentrated under reduced pressure. The residue was purified by argentation flash chromatography (SiO_2_·AgNO_3_, pentane) to give **21** as a colorless oil (45 mg, 0.098 mmol, 86%). IR (neat) ν_max_ 3005, 2920, 2853, 1739, 1458, 1370, 1303, 1217, 1071, 967, 719, 603, 555, 545 cm^–1^; ^1^H NMR (CDCl_3_, 300 MHz) δ 5.47–5.24 (4H, m), 2.12–1.89 (8H, m), 1.42–1.01 (43H, m), 0.95 (3H, t, *J* = 7.5 Hz), 0.88 (3H, t, *J* = 6.7 Hz), 0.83 (3H, d, *J* = 6.4 Hz); ^13^C NMR, DEPT (CDCl_3_, 75 MHz) δ 131.6 (CH), 130.1 (CH), 130.0 (CH), 129.5 (CH), 37.3 (CH_2_), 32.9 (CH_2_), 32.1 (CH), 30.2 (CH_2_), 29.9 (CH_2_), 29.9 (CH_2_), 29.9 (CH_2_), 29.8 (CH_2_), 29.7 (CH_2_), 29.7 (CH_2_), 29.5 (CH_2_), 29.5 (CH_2_), 29.5 (CH_2_), 27.4 (CH_2_), 27.3 (CH_2_), 22.9 (CH_2_), 20.7 (CH_2_), 19.9 (CH_3_), 14.6 (CH_3_), 14.3 (CH_3_); EIMS *m*/*z* 460 ([M]^+^, 4), 96 (67), 83 (60), 82 (83), 81 (56), 69 (78), 67 (52), 57 (80), 55 (100), 43 (73), 41 (55). HRCIPMS *m*/*z* 459.49271 [M – H]^+^ (calcd for C_33_H_63_, 459.4924); GC *I* 3210.

#### *cis*,*cis*-3,4,13,14-Bismethylene-24-methyldotriacontane (**4**)

Cyclopropanation was performed according to a modified procedure of Pragliola et al.^52^ A solution of AlEt_3_ (1 M in hexane) was added to a stirred solution of diene **21** (25 mg, 0.054 mmol, 1.0 equiv) and CH_2_I_2_ (13 μL, 0.163 mmol, 3.0 equiv) in CH_2_Cl_2_ (1 mL). After stirring for 17 h at rt, NaF (42 mg, 0.540 mmol, 1.0 equiv) and H_2_O (1 mL) were added to the reaction mixture. A nonstirrable jelly formed, which was dissolved by addition of HCl (1 M, 1 mL). The resulting mixture was stirred for a further 10 min and extracted with CH_2_Cl_2_ (2 × 5 mL). The combined organic phases were dried over MgSO_4_ and concentrated under reduced pressure. The residue was purified by argentation flash chromatography (SiO_2_·AgNO_3_, pentane) to give **4** as a colorless oil (25 mg, 0.051 mmol, 95%). IR (neat) ν_max_ 3060, 2990, 2921, 2852, 1461, 1374, 1305, 1020, 848, 816, 721, 680, 572, 549 cm^–1^; ^1^H NMR (CDCl_3_, 500 MHz) δ 1.45–1.02 (51H, m), 0.98 (3H, t, *J* = 7.3 Hz), 0.88 (3H, t, *J* = 7.0 Hz), 0.84 (3H, d, *J* = 6.6 Hz), 0.72–0.59 (4H, m), 0.59–0.53 (2H, m), −0.33 (2H, dd, *J* = 9.5, 5.3 Hz); ^13^C NMR, DEPT (CDCl_3_, 126 MHz) δ 37.1 (CH), 32.8 (CH_2_), 32.0 (CH_2_), 30.3 (CH_2_), 30.3 (CH_2_), 30.1 (CH_2_), 29.8 (CH_2_), 29.7 (CH_2_), 29.7 (CH_2_), 29.4 (CH_2_), 28.8 (CH_2_), 28.7 (CH_2_), 27.1 (CH_2_), 22.7 (CH_2_), 21.9 (CH_2_), 19.7 (CH_3_), 17.7 (CH), 16.0 (CH), 15.8 (CH), 14.5 (CH_3_), 14.1 (CH_3_), 10.9 (CH_2_), 10.7 (CH_2_); EIMS *m*/*z* 488 (4, [M]^+^), 432 (1), 194 (5), 97 (61), 96 (81), 95 (58), 83 (76), 82 (88), 69 (78), 57 (75), 81 (60), 55 (100), 43 (54); HRCIPMS *m*/*z* 487.52402 [M – H]^+^ (calcd for C_35_H_67_, 487.52373); GC *I* 3446.

#### (3*E*,13*E*)-24-Methyldotriaconta-3,13-diyne (**22**)

Diene **10** (52 mg, 0.114 mmol, 1.0 equiv) and HMPA (1 mL) were dissolved in THF (1 mL). The mixture was cooled to −40 °C, NH_3_ was condensed into it, and a Na piece (26 mg, 1.138 mmol, 10 equiv) was added. After a dark blue color appeared, the mixture was allowed to reflux, releasing most of the NH_3_.^29^ After 1 h, the mixture was warmed to rt, allowing the remaining NH_3_ to evaporate completely. H_2_O (10 mL) was then added and extracted with pentane (3 × 10 mL). The combined organic phases were dried over MgSO_4_ and concentrated under reduced pressure. The residue was purified by argentation flash chromatography (SiO_2_·AgNO_3_, pentane) to give **22** as a colorless oil (1 mg, 0.002 mmol, 2%). IR (neat) ν_max_ 2961, 2922, 2872, 2852, 1471, 1377, 965, 722 cm^–1^; ^1^H NMR (CDCl_3_, 600 MHz) δ 5.49–5.32 (m, 4H), 2.06–1.91 (m, 9H), 1.44–1.13 (m, 56H), 1.12–1.03 (m, 3H), 0.96 (t, *J* = 7.4 Hz, 2H), 0.88 (t, *J* = 7.0 Hz, 5H), 0.83 (d, *J* = 6.6 Hz, 5H); ^13^C NMR, DEPT (CDCl_3_, 150 MHz) δ 132.0 (CH), 130.5 (CH), 130.5 (CH), 129.6 (CH), 37.3 (CH_2_), 32.9 (CH), 32.8 (CH_2_), 32.7 (CH_2_), 32.1 (CH_2_), 30.2 (CH_2_), 29.9 (CH_2_), 29.8 (CH_2_), 29.7 (CH_2_), 29.7 (CH_2_), 29.5 (CH_2_), 29.3 (CH_2_), 29.2 (CH_2_), 27.4 (CH_2_), 27.3 (CH_2_), 25.8 (CH_2_), 22.9 (CH_2_), 19.9 (CH_3_), 14.3 (CH_3_), 14.2 (CH_3_); EIMS *m*/*z* 460 ([M]^+^, 4), 96 (70), 83 (63), 82 (91), 81 (58), 69 (87), 68 (55), 57 (83), 55 (100), 43 (74), 41 (57); HRCIPMS *m*/*z* 459.49271 [M – H]^+^ (calcd for C_33_H_63_, 459.4924); GC *I* 3209.

#### *trans*,*trans*-3,4,13,14-Bismethylene-24-methyldotriacontane (**23**)

This compound was prepared via the cyclopropanation microderivatization method described above. IR (neat) ν_max_ 3071, 2959, 2921, 2872, 2852, 1468, 1376, 1019, 900, 723 cm^–1^; EIMS *m*/*z* 488 (4, [M]^+^), 432 (1), 194 (5), 97 (61), 96 (81), 95 (58), 83 (76), 82 (88), 69 (78), 57 (75), 81 (60), 55 (100), 43 (54); HRCIPMS *m*/*z* 487.52402 [M – H]^+^ (calcd for C_35_H_67_, 487.52373); GC *I* 3379.

## Data Availability

Original NMR data of **A** and compounds **4**, **10**, **13**, **16**, and **20**–**22** can be found in the Leopard data repository of TU Branschweig under the address 10.24355/dbbs.084-202310270956-0.

## Supporting Information Available

Mass spectra, NMR spectra of sarekensane and the synthetic compounds(PDF)
