# Supplementary material for: Cyclopropane Hydrocarbons from the Springtail Vertagopus sarekensis—A New Class of Cuticular Lipids from Arthropods
Source: J Nat Prod. 2023 Nov 13;87(1):85–97. doi: 10.1021/acs.jnatprod.3c00789 (PMC10825826; doi:10.1021/acs.jnatprod.3c00789)
Supplement: Supplementary file 1 — np3c00789_si_001.pdf [file np3c00789_si_001.pdf]

Supporting information

Table of Contents

1 Mass Spectra of the Hydrogenation Products……………………………………...………………...1

2 NMR Spectra of Natural Compound **A**…......…………………………………………………………...5

3 NMR Spectra of Synthetic Compounds …………………………………………………………...…...8

1 Mass Spectra of the Hydrogenation Products

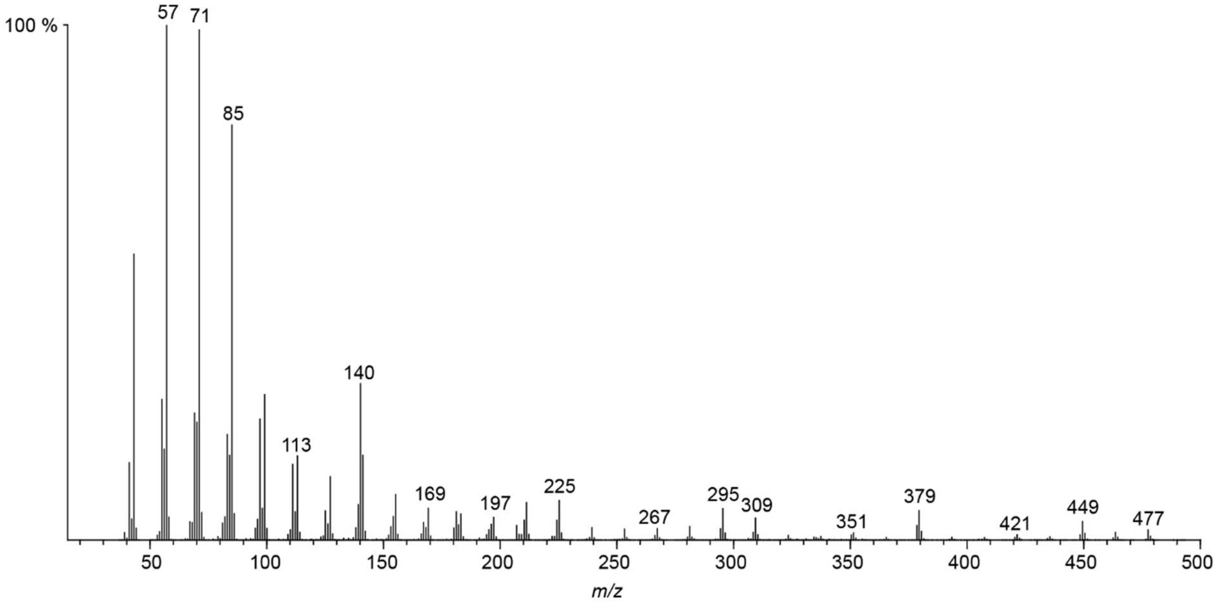

Figure S1. Mass spectrum of peak **B** of the hydrogenated sample of compound **A**

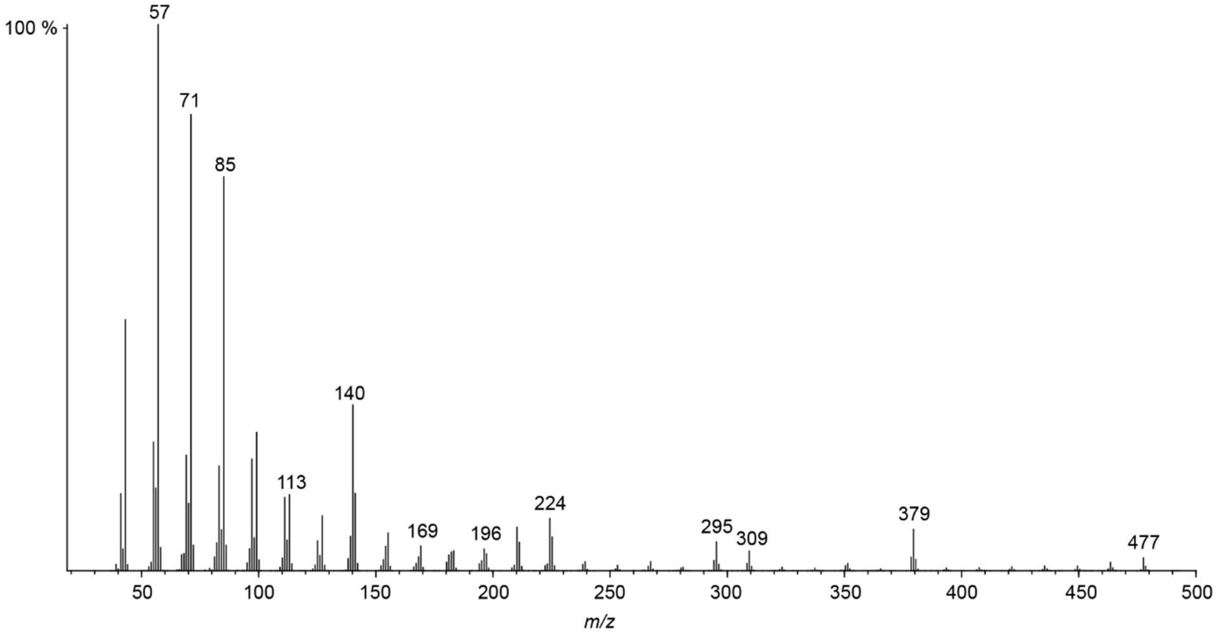

Figure S2. Mass spectrum of peak **D** of the hydrogenated sample of compound **A**

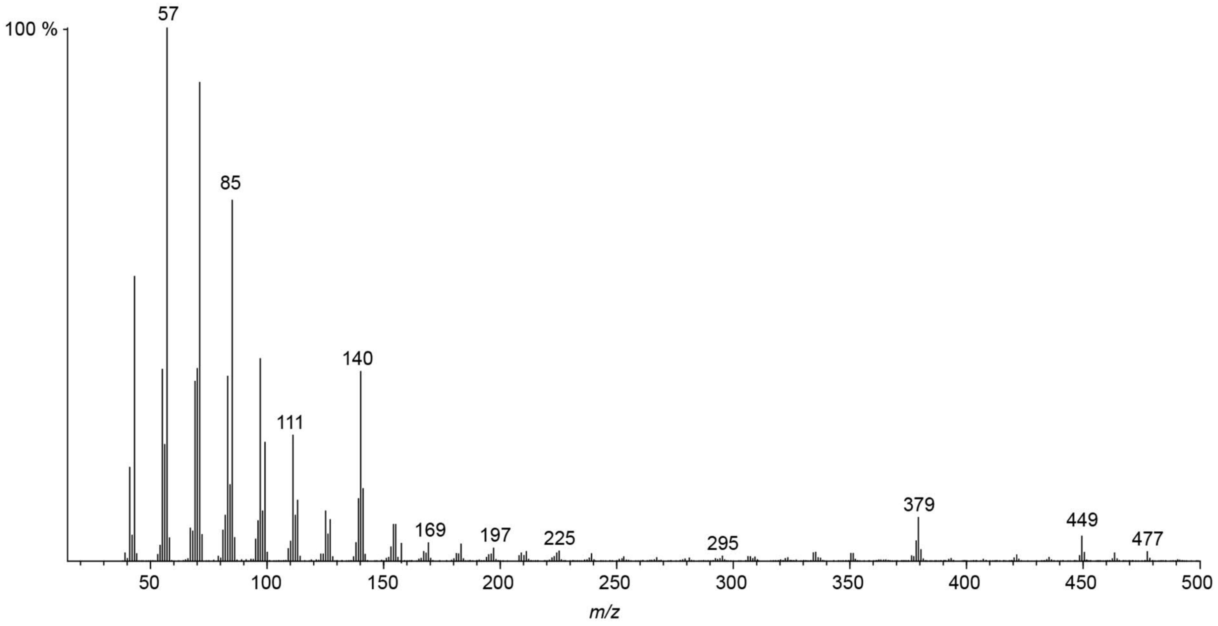

Figure S3. Mass spectrum of peak **E** of the hydrogenated sample of compound **A**

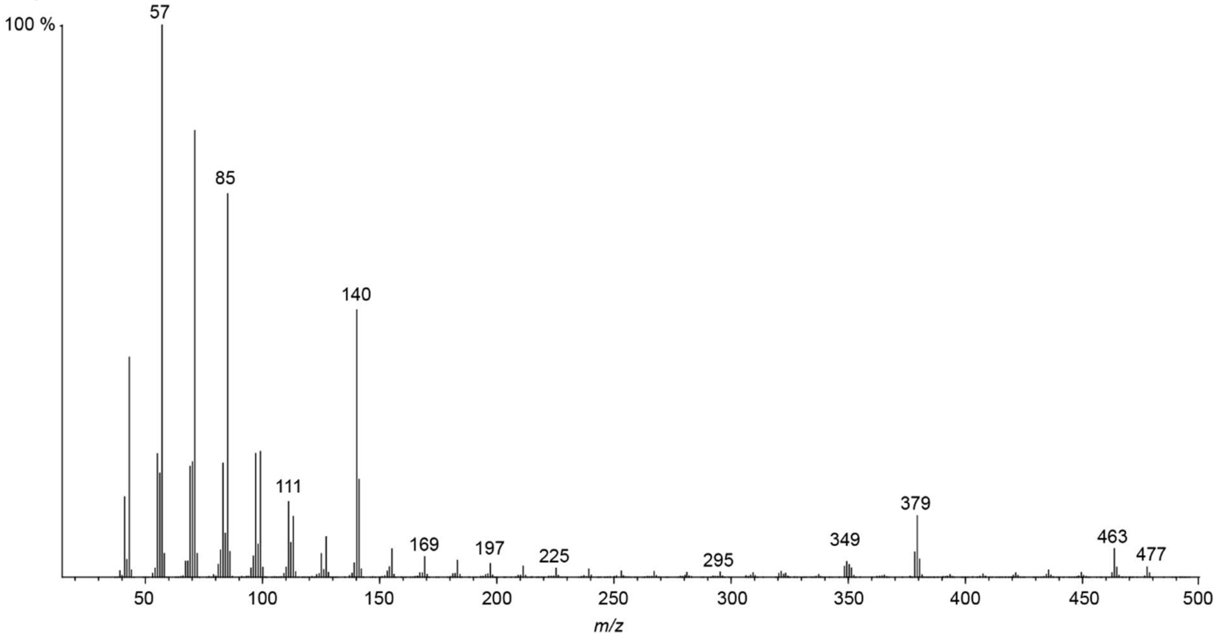

Figure S4. Mass spectrum of peak **F** of the hydrogenated sample of compound **A**

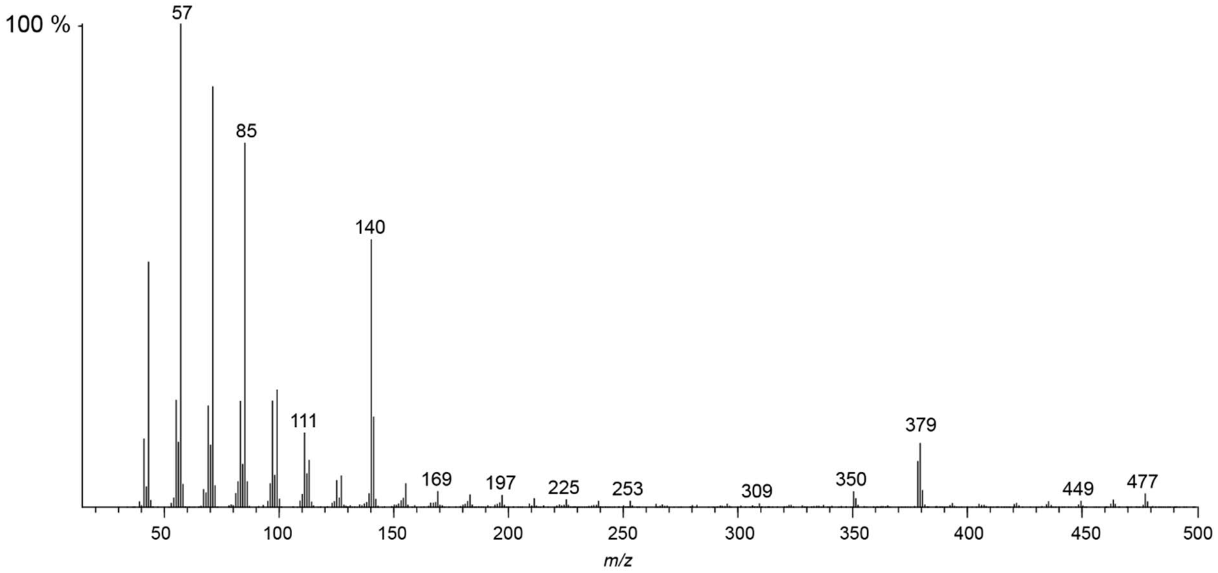

Figure S5. Mass spectrum of peak **G** of the hydrogenated sample of compound **A**.

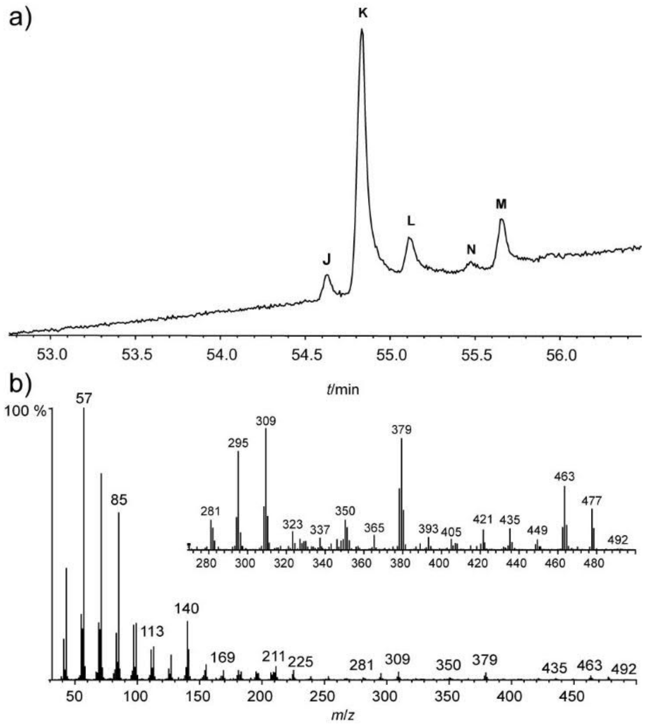

Figure S6. a) TIC of synthetic product **4** after hydrogenation conditions (Pd/C, H_2). b) Mass spectrum of peak **K**.

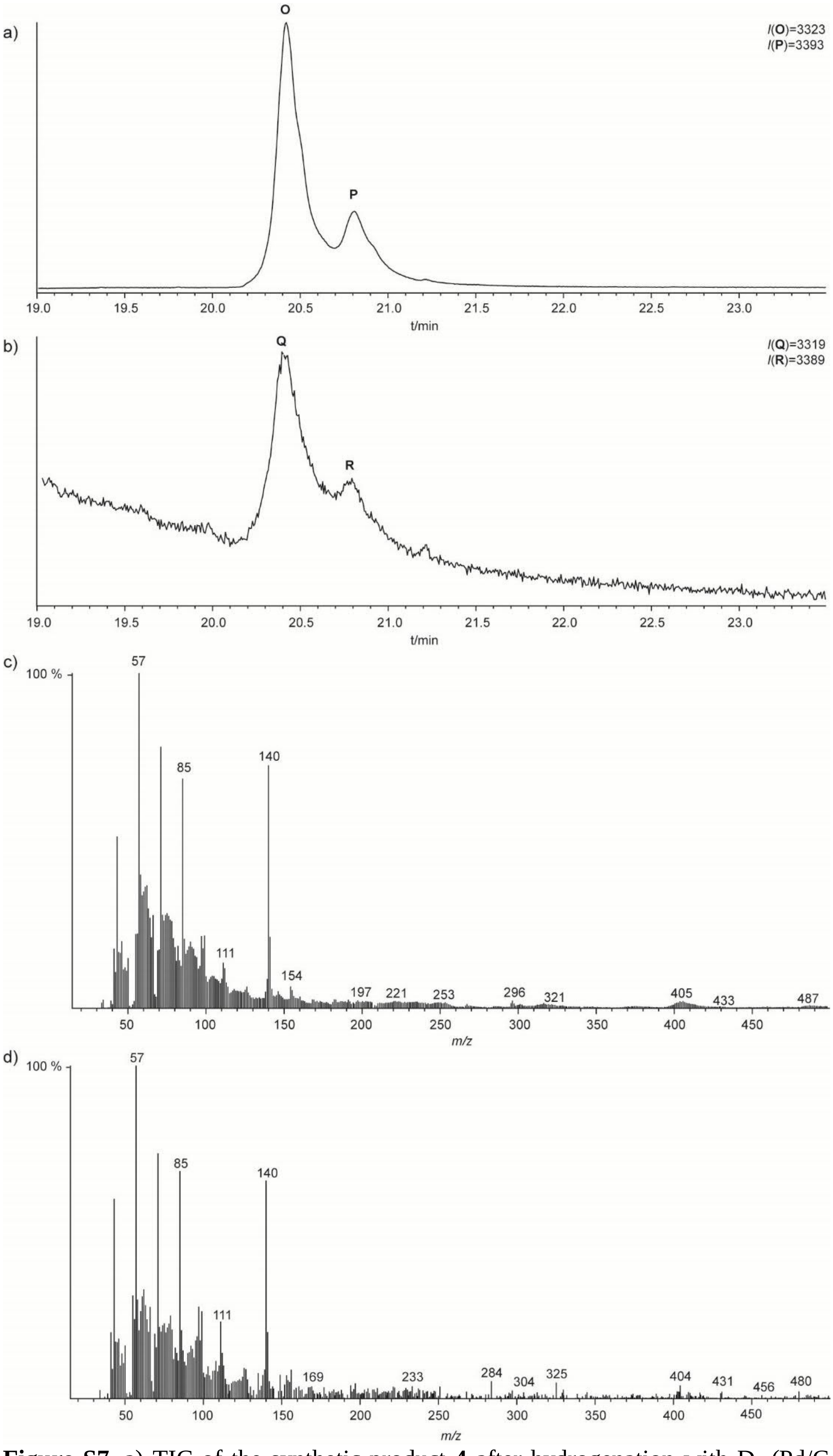

Figure S7. a) TIC of the synthetic product **4** after hydrogenation with D_2 (Pd/C, D_2); b) TIC of the natural compound **A** after hydrogenation with D_2 (Pd/C, D_2); c) Mass spectrum of peak **O**; d) Mass spectrum of peak **Q**.

2 NMR spectra of Natural Compound A

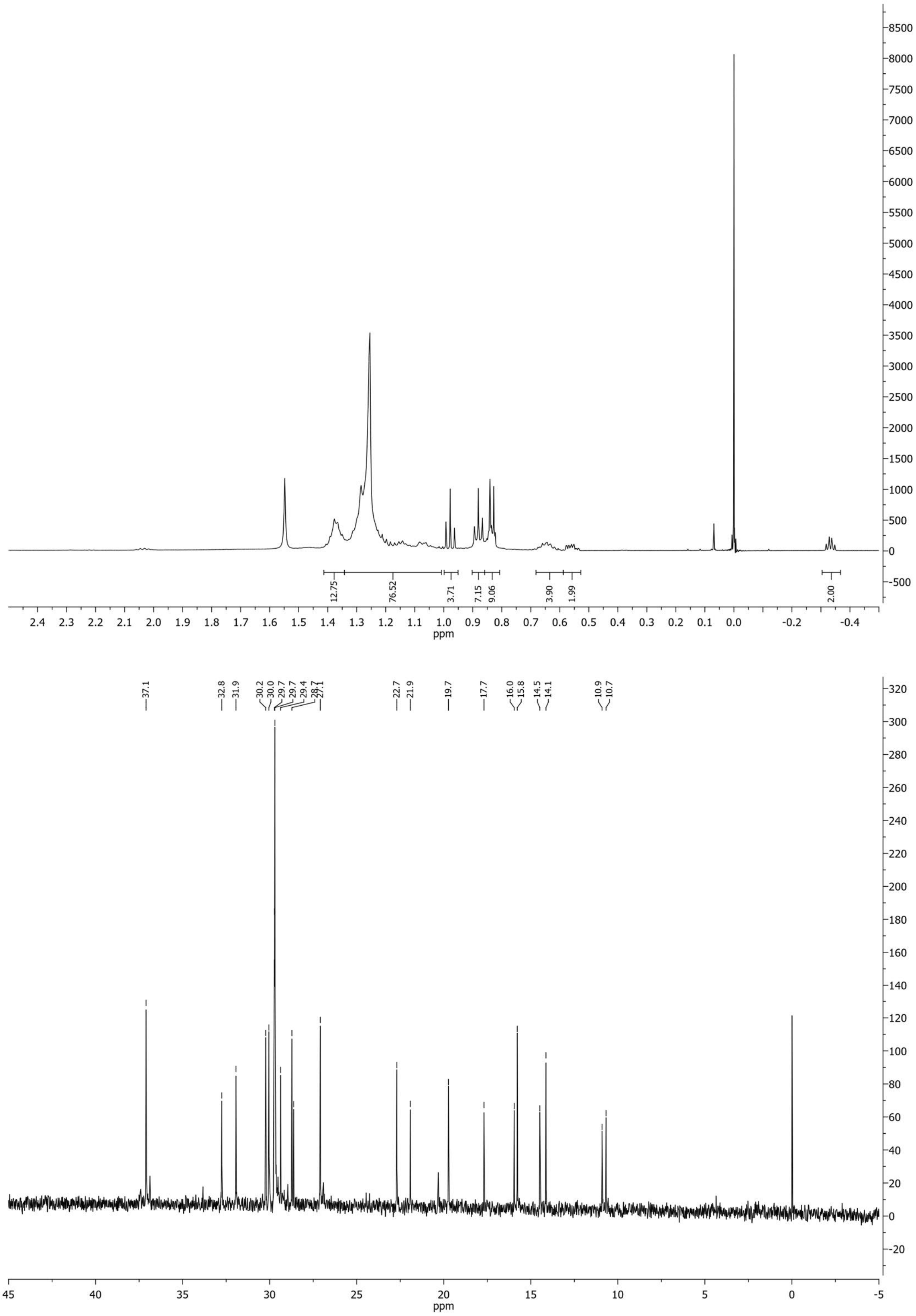

Figure S8. ^{1}H-NMR ($CDCl_3$, 500 MHz) and ^{13}C-NMR ($CDCl_3$, 125 MHz) of compound **A**.

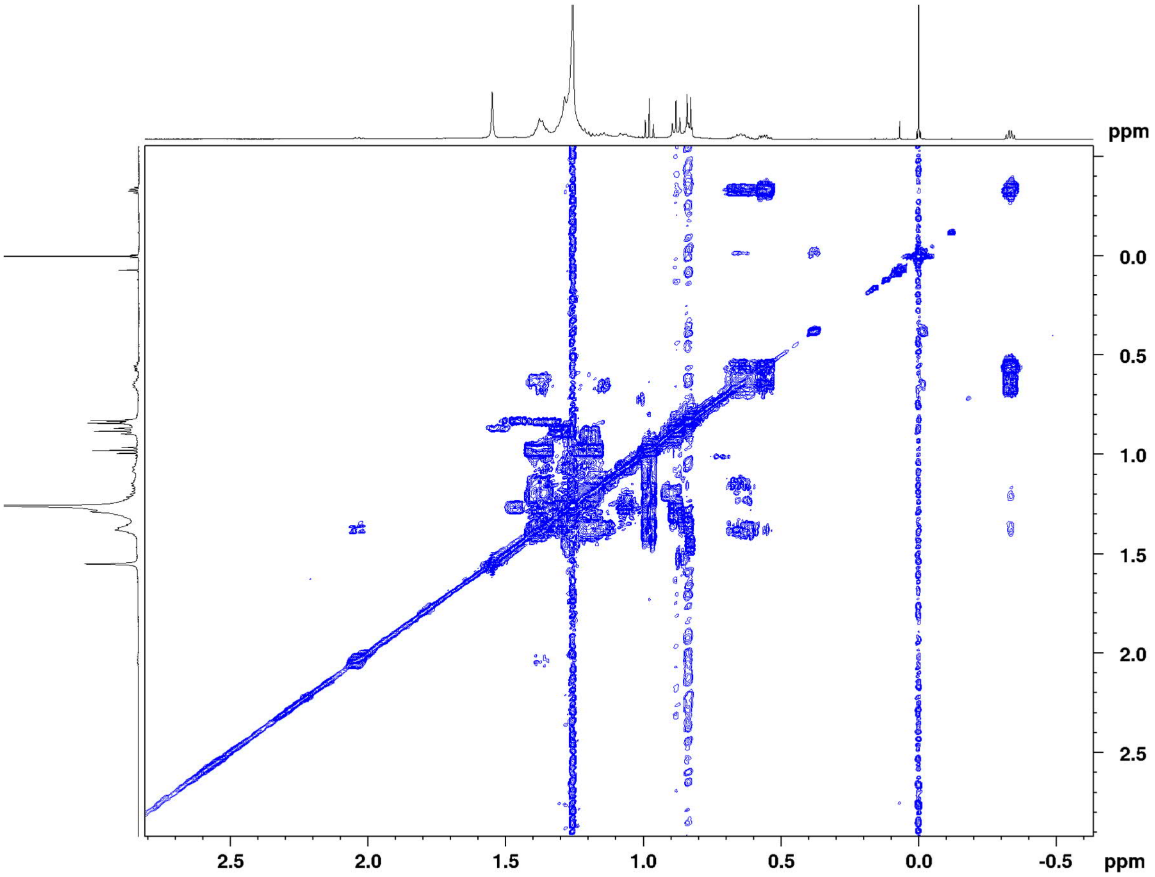

Figure S9. COSY ($CDCl_3$, 500 MHz) of compound **A**.

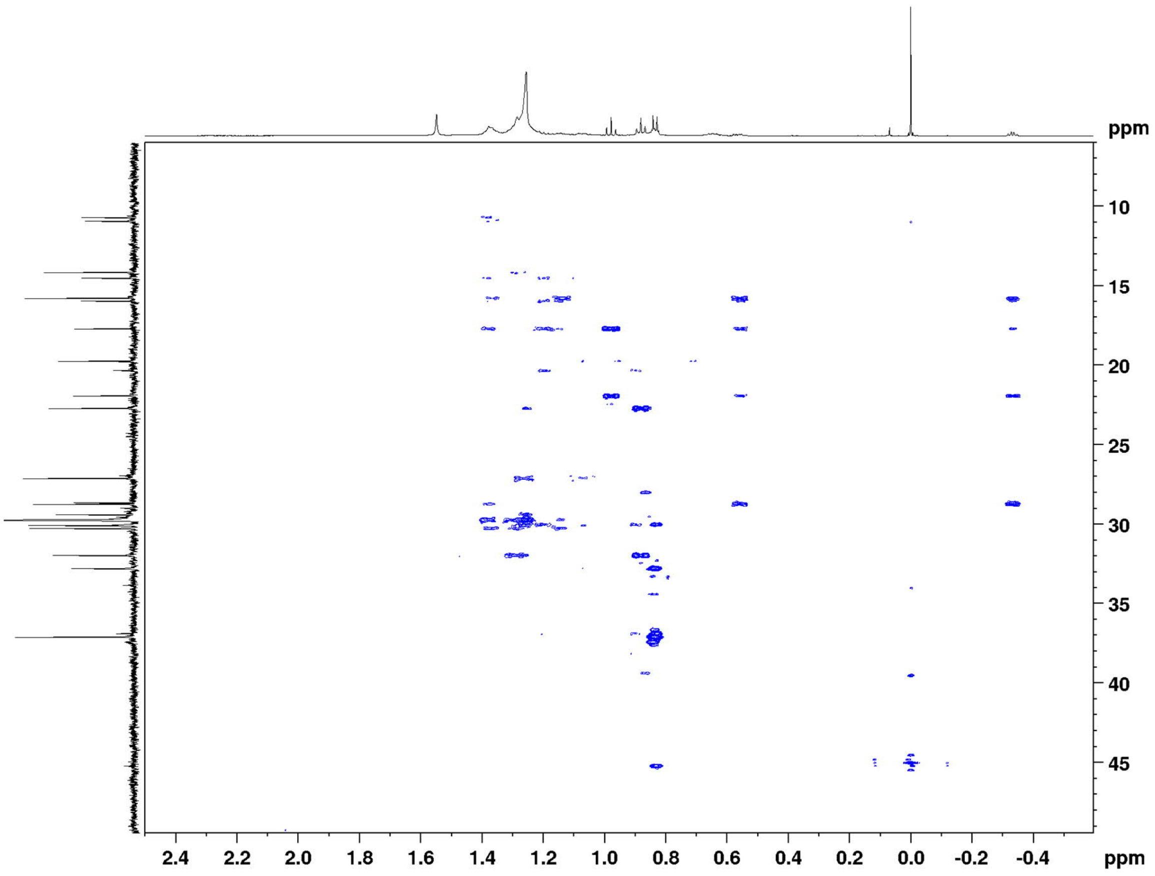

Figure S10. HMBC ($CDCl_3$, 500/125 MHz) of compound **A**.

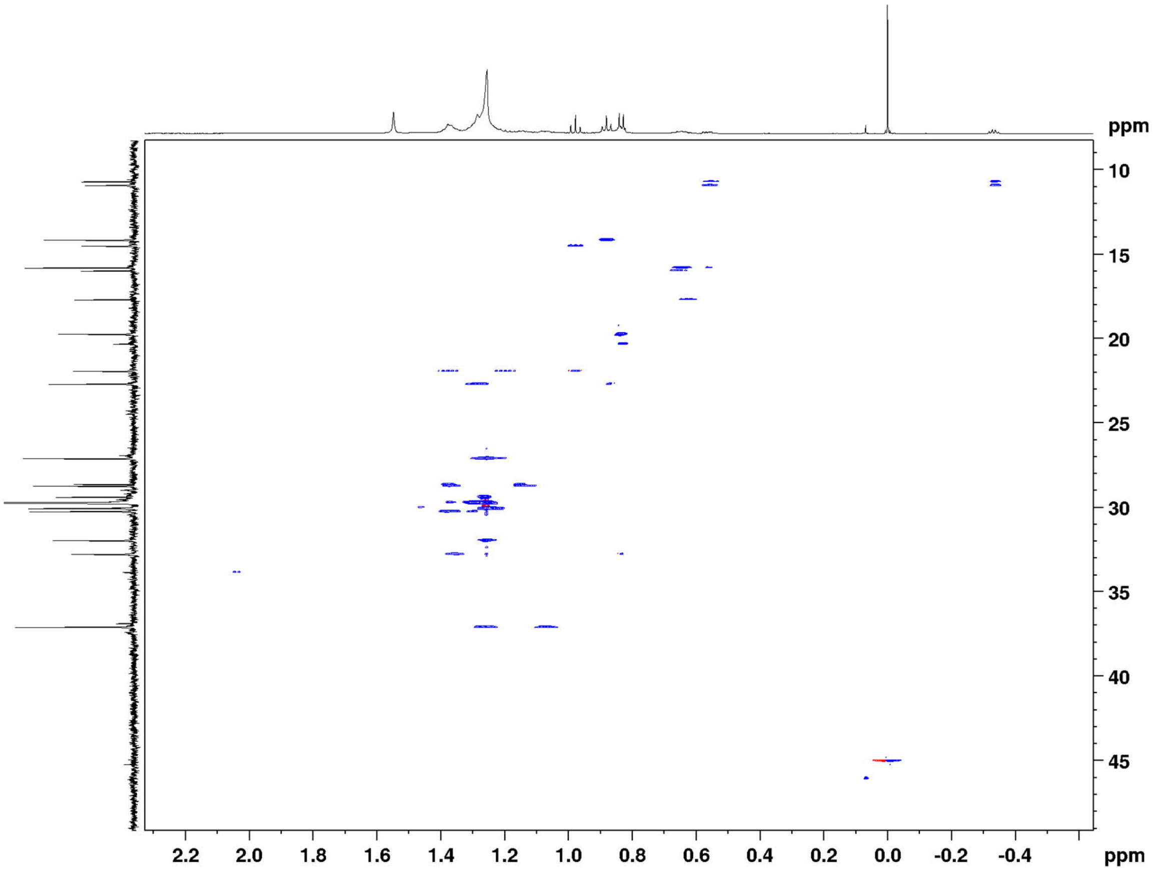

Figure S11. HSQC ($CDCl_3$, 500/125 MHz) of compound **A**.

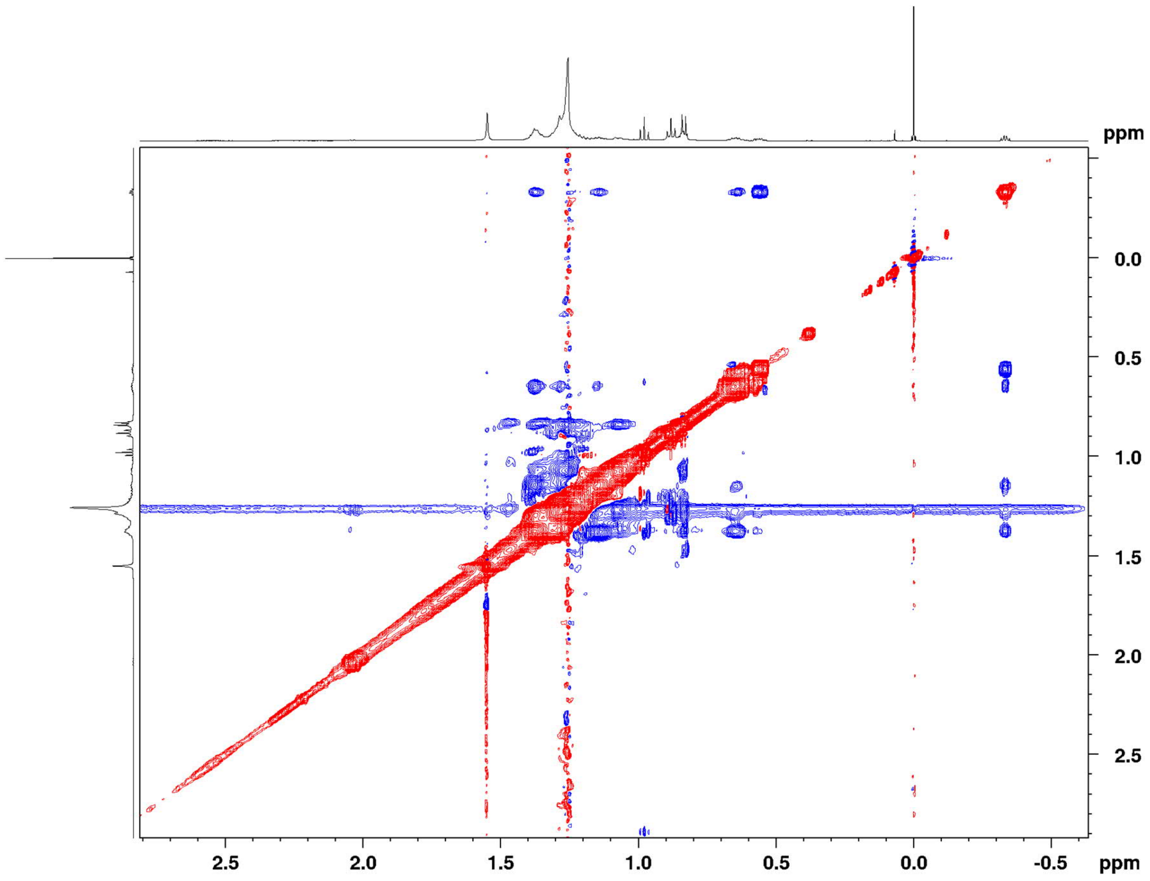

Figure S12. NOESY ($CDCl_3$, 500 MHz) of compound **A**.

3 NMR Spectra of Synthetic Compounds

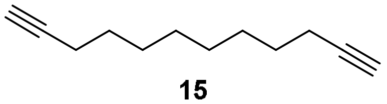

15

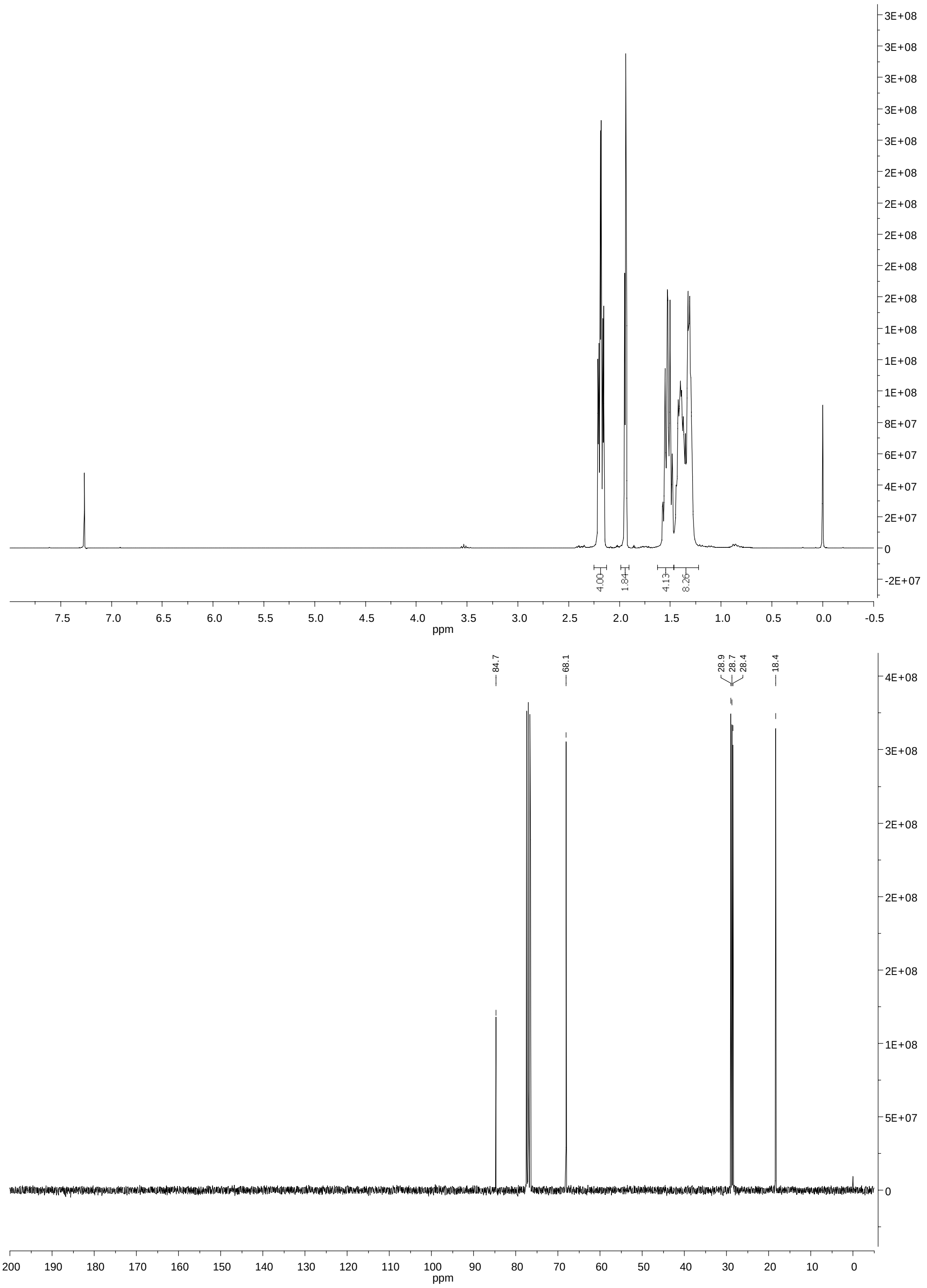

Figure S13. ^{1}H-NMR ($CDCl_3$, 300 MHz) and ^{13}C-NMR ($CDCl_3$, 75 MHz) of **15**.

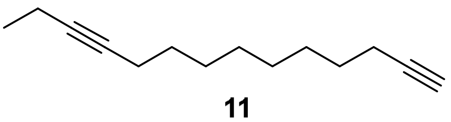

11

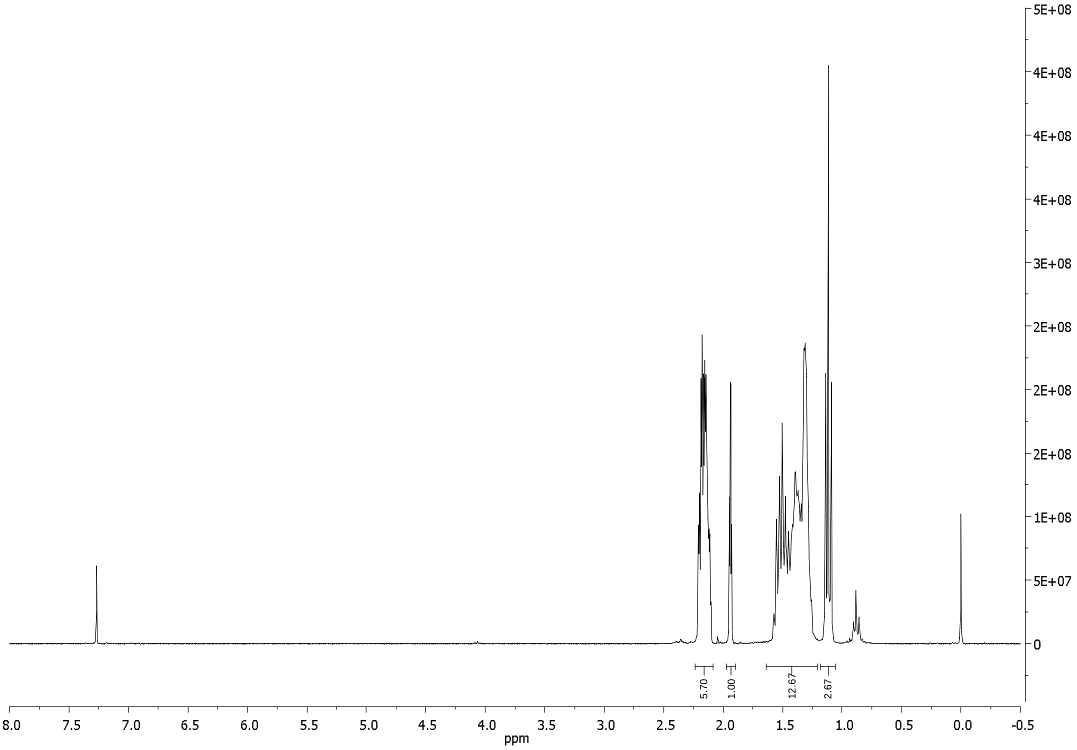

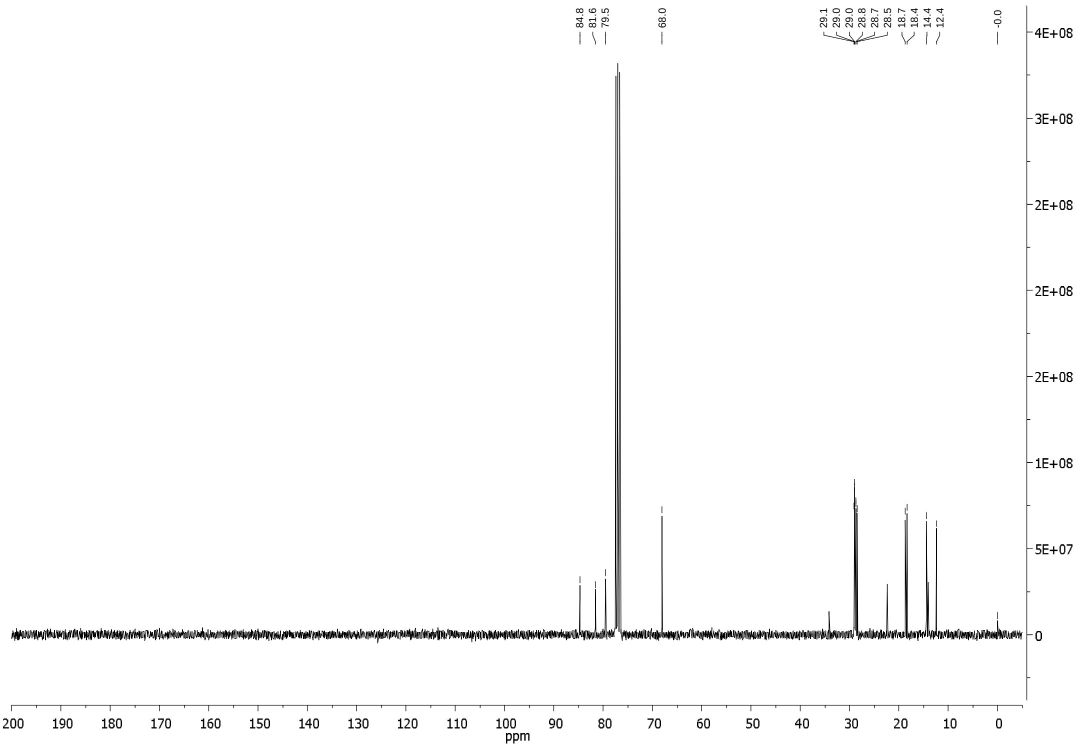

Figure S14. ^{1}H-NMR ($CDCl_3$, 300 MHz) and ^{13}C-NMR ($CDCl_3$, 75 MHz) of **11**.

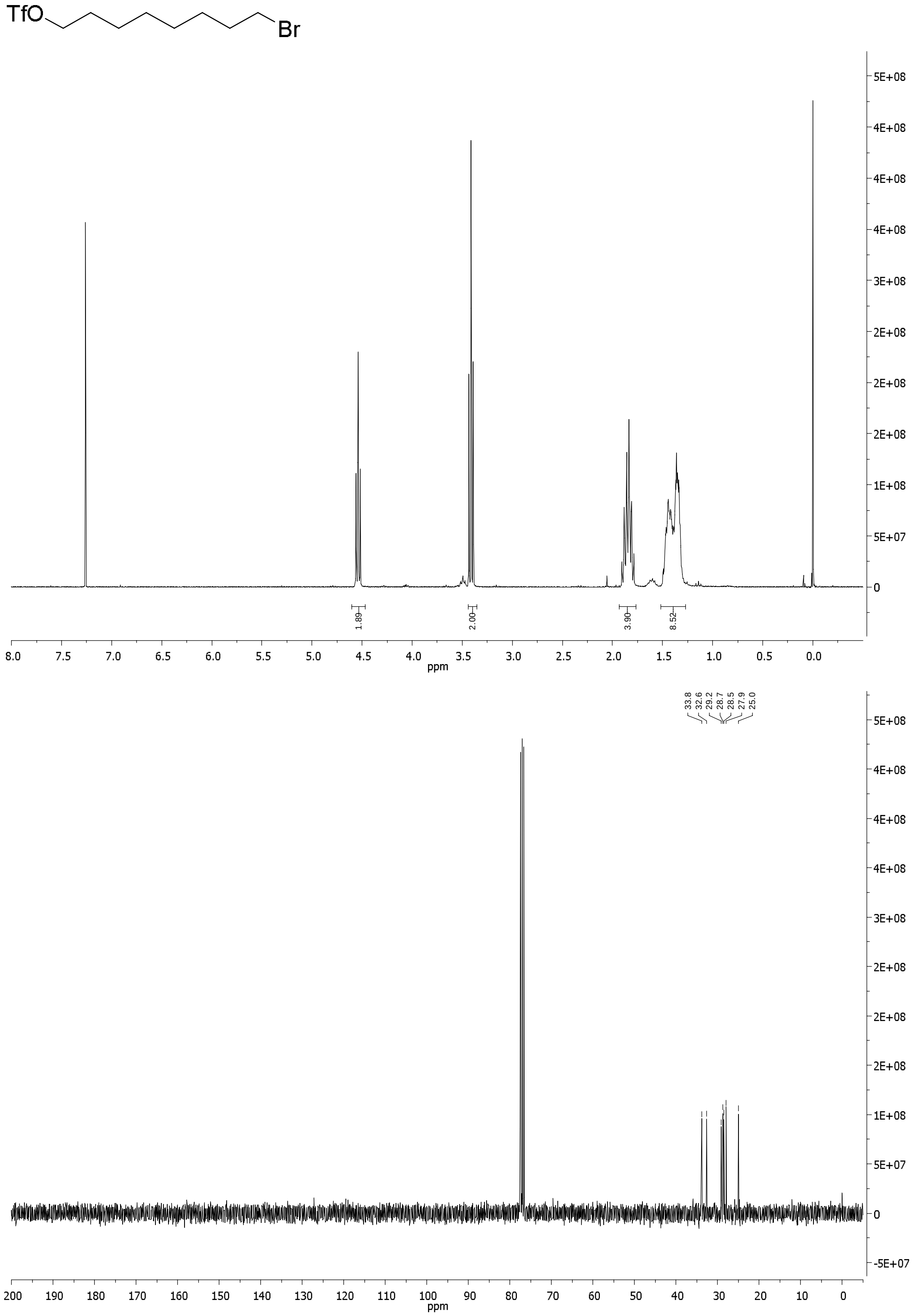

Figure S15. ^{1}H-NMR ($CDCl_3$, 300 MHz) and ^{13}C-NMR ($CDCl_3$, 75 MHz) of 8-bromooctyl trifluoromethanesulfonate.

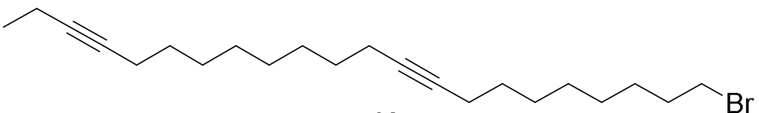

16

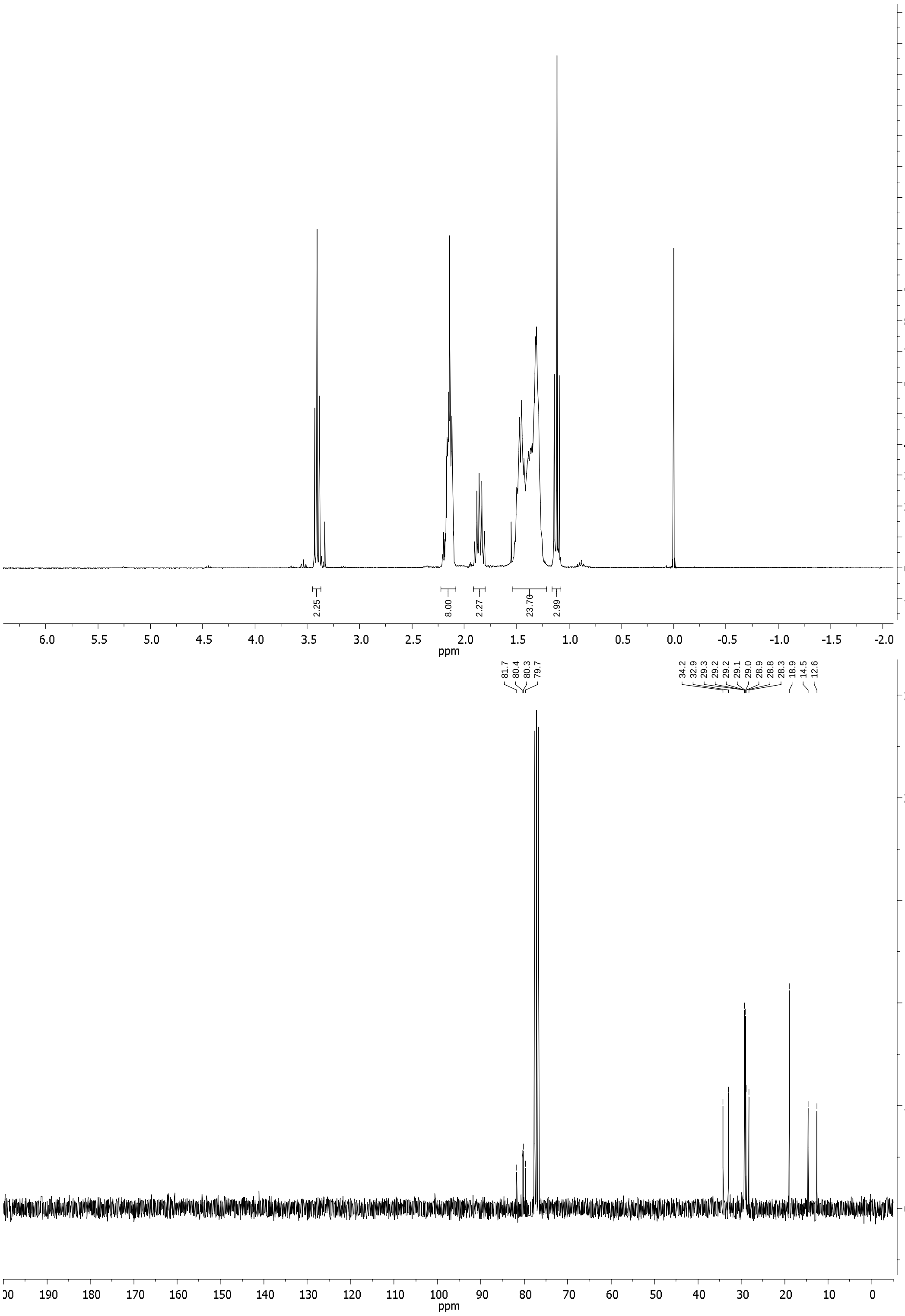

Figure S16. ^{1}H-NMR ($CDCl_3$, 300 MHz) and ^{13}C-NMR ($CDCl_3$, 75 MHz) of **16**.

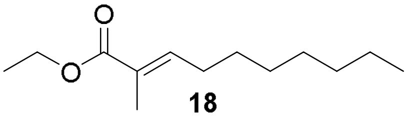

18

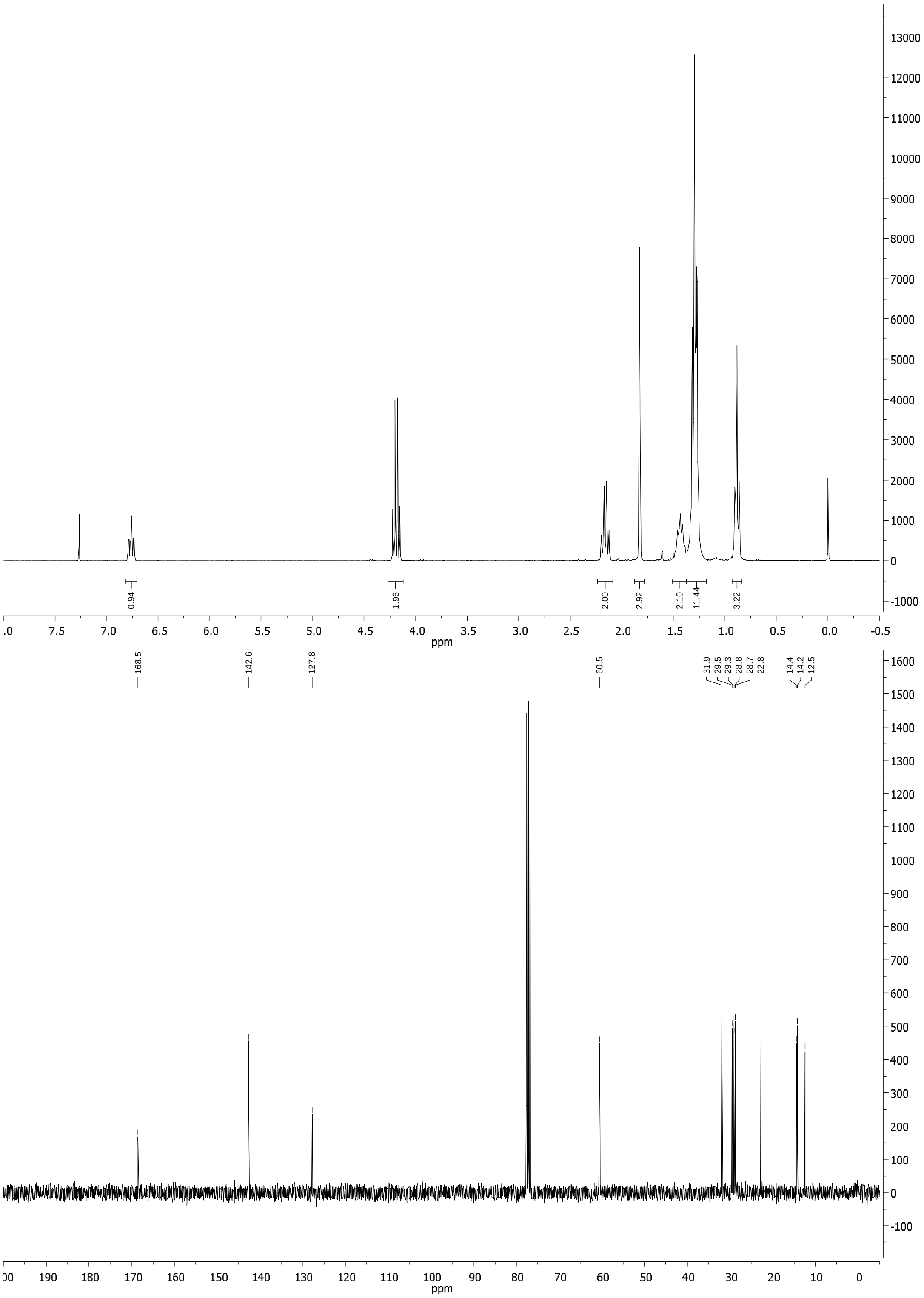

Figure S17. ^{1}H-NMR ($CDCl_3$, 300 MHz) and ^{13}C-NMR ($CDCl_3$, 75 MHz) of **18**.

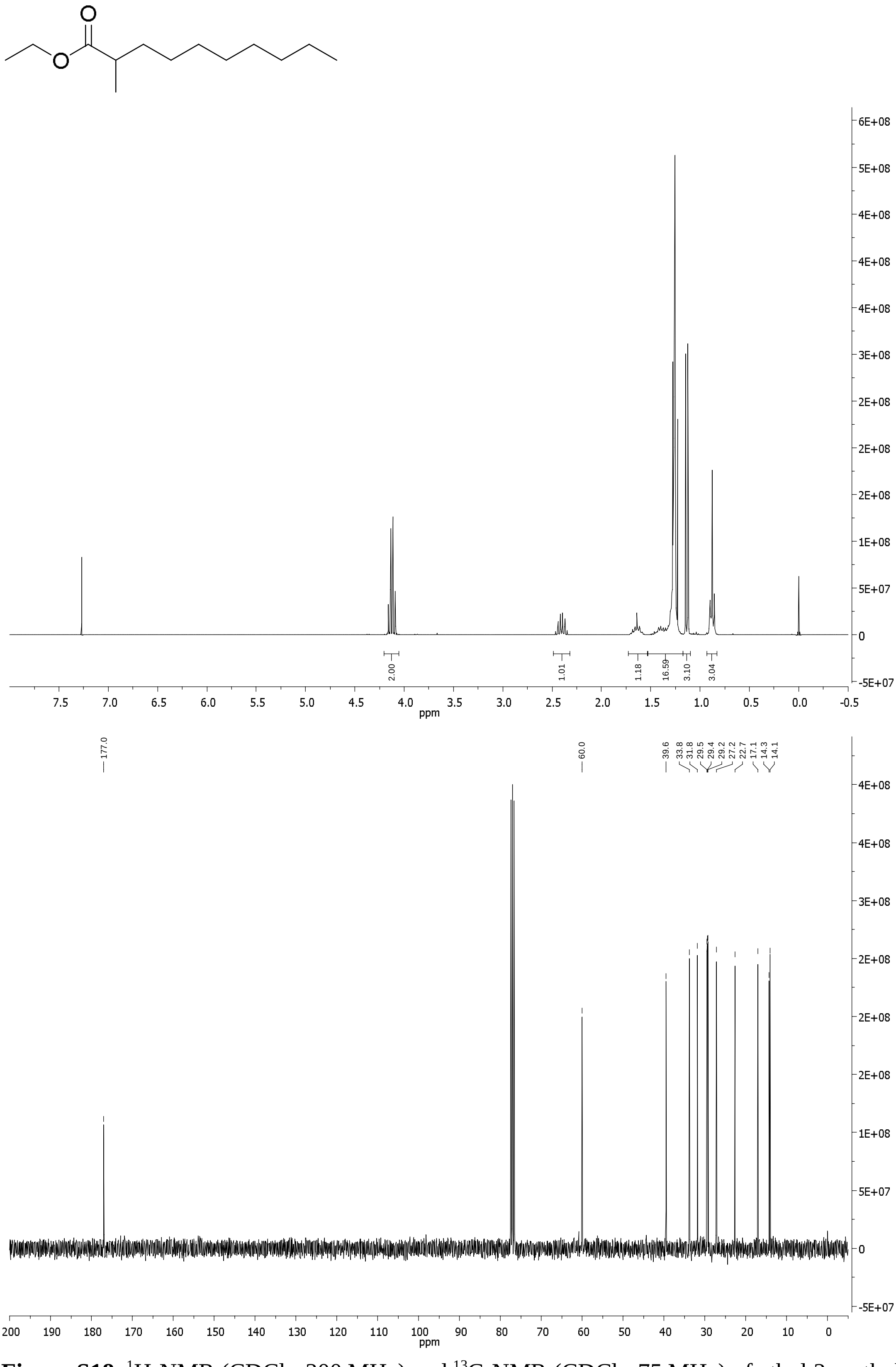

Figure S18. ^{1}H-NMR ($CDCl_3$, 300 MHz) and ^{13}C-NMR ($CDCl_3$, 75 MHz) of ethyl 2-methyldecanoate.

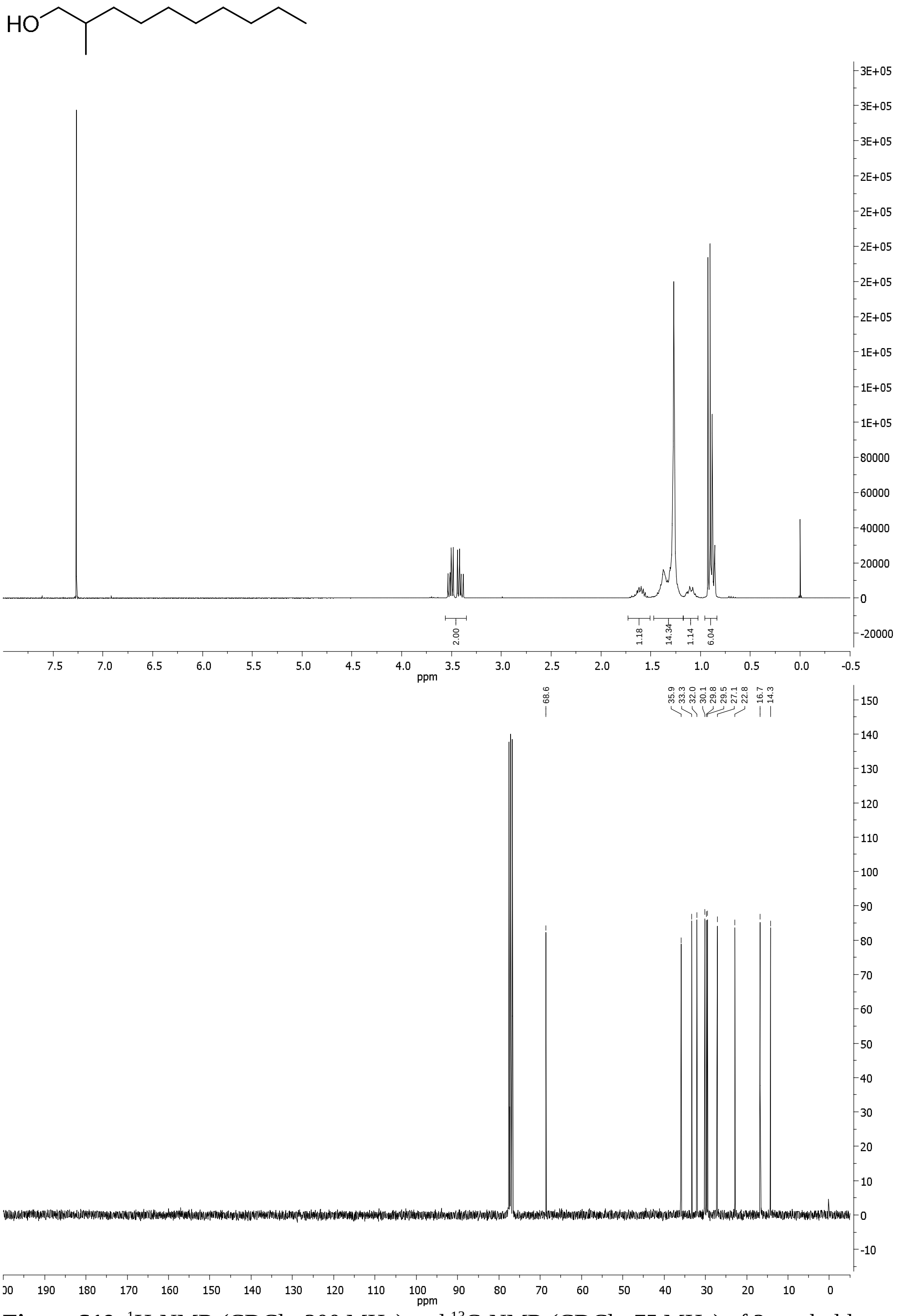

Figure S19. ^{1}H-NMR ($CDCl_3$, 300 MHz) and ^{13}C-NMR ($CDCl_3$, 75 MHz) of 2-methyldecan-1-ol.

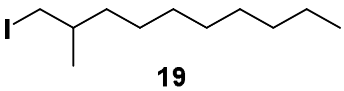

19

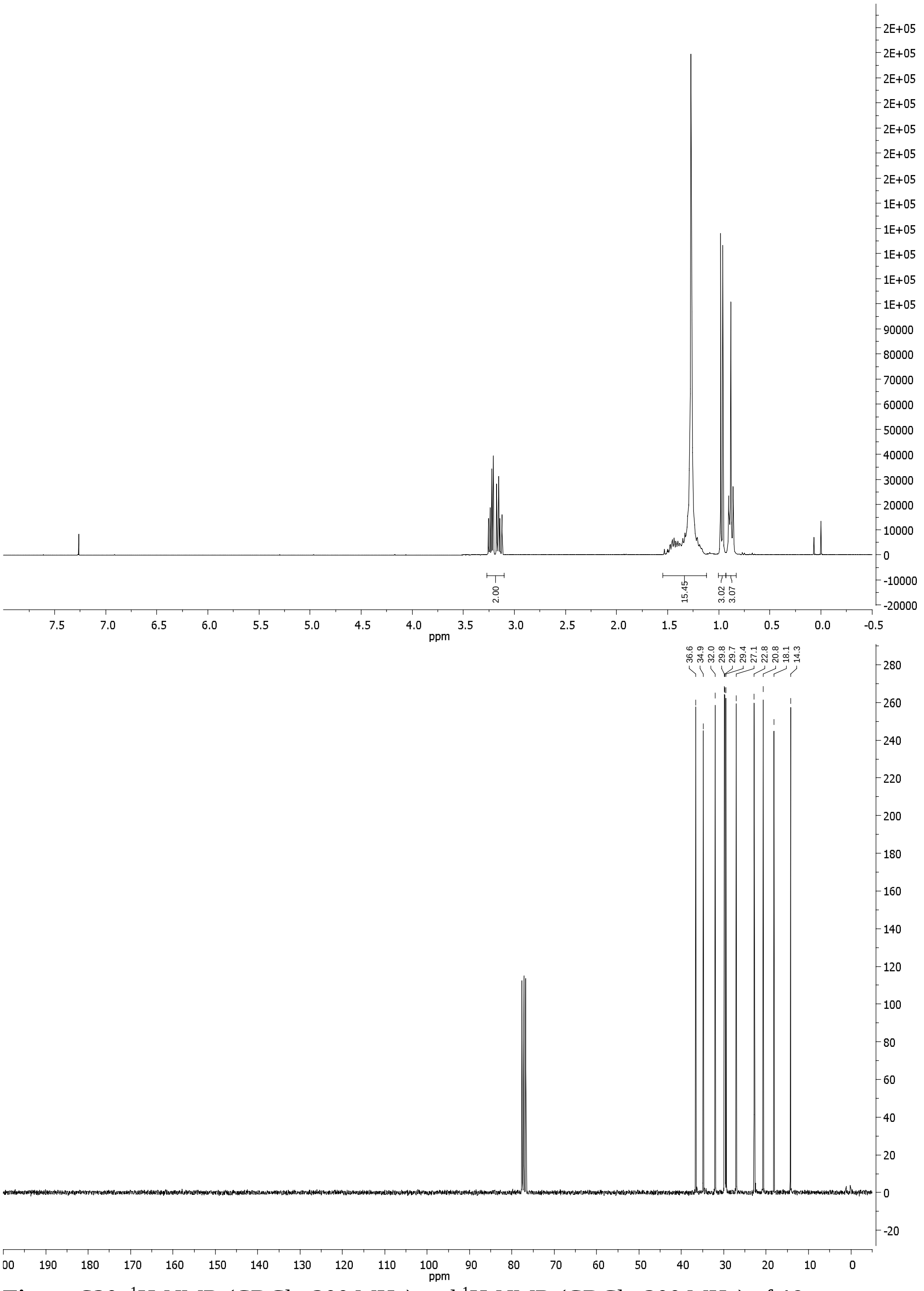

Figure S20. ^{1}H-NMR ($CDCl_3$, 300 MHz) and ^{1}H-NMR ($CDCl_3$, 300 MHz) of **19**.

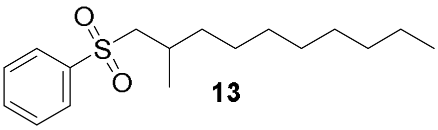

13

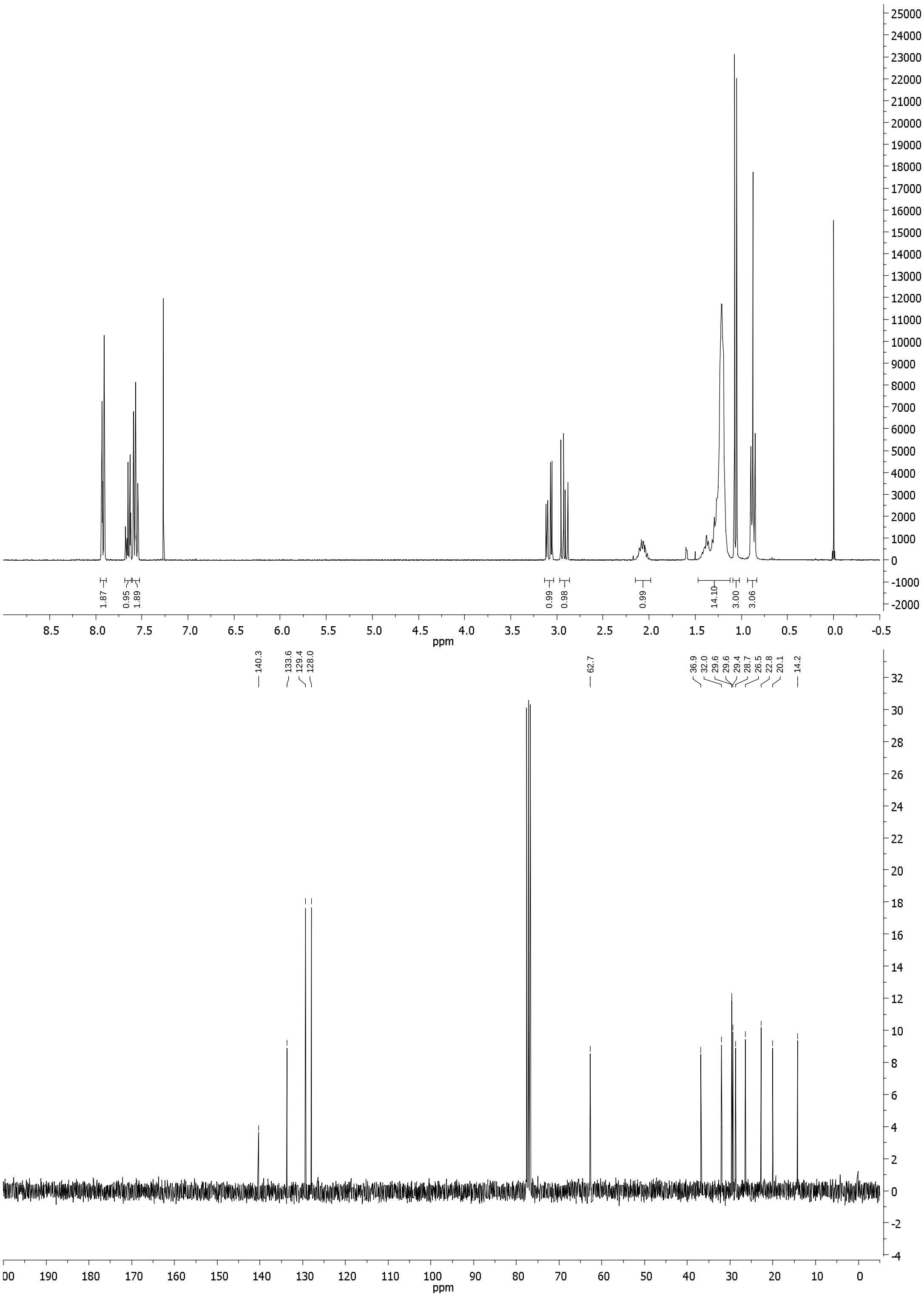

Figure S21. ^{1}H-NMR ($CDCl_3$, 300 MHz) and ^{13}C-NMR ($CDCl_3$, 75 MHz) of **13**.

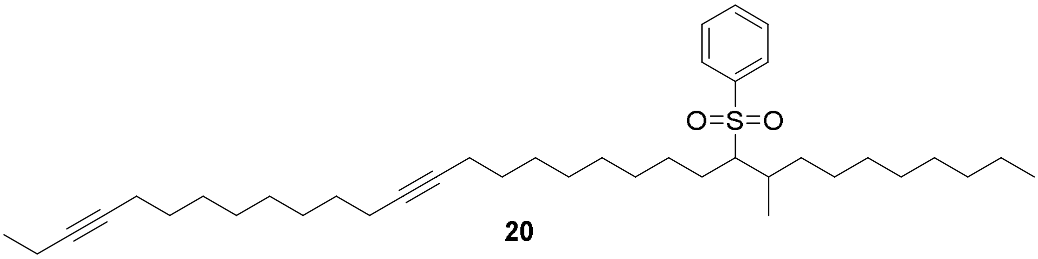

20

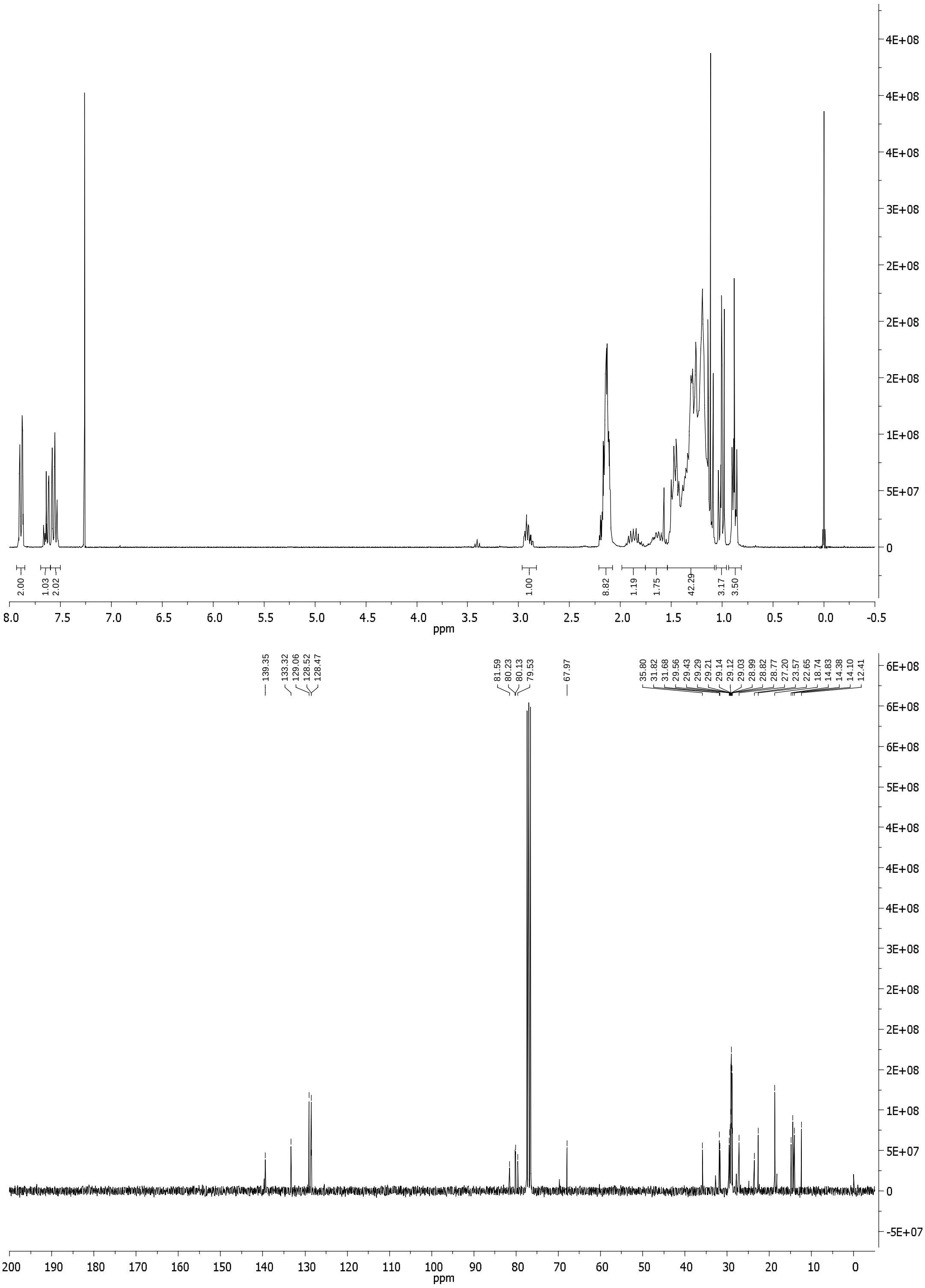

Figure S22. ^{1}H-NMR ($CDCl_3$, 300 MHz) and ^{13}C-NMR ($CDCl_3$, 75 MHz) of **20**.

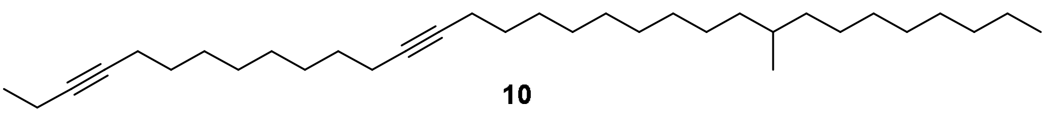

10

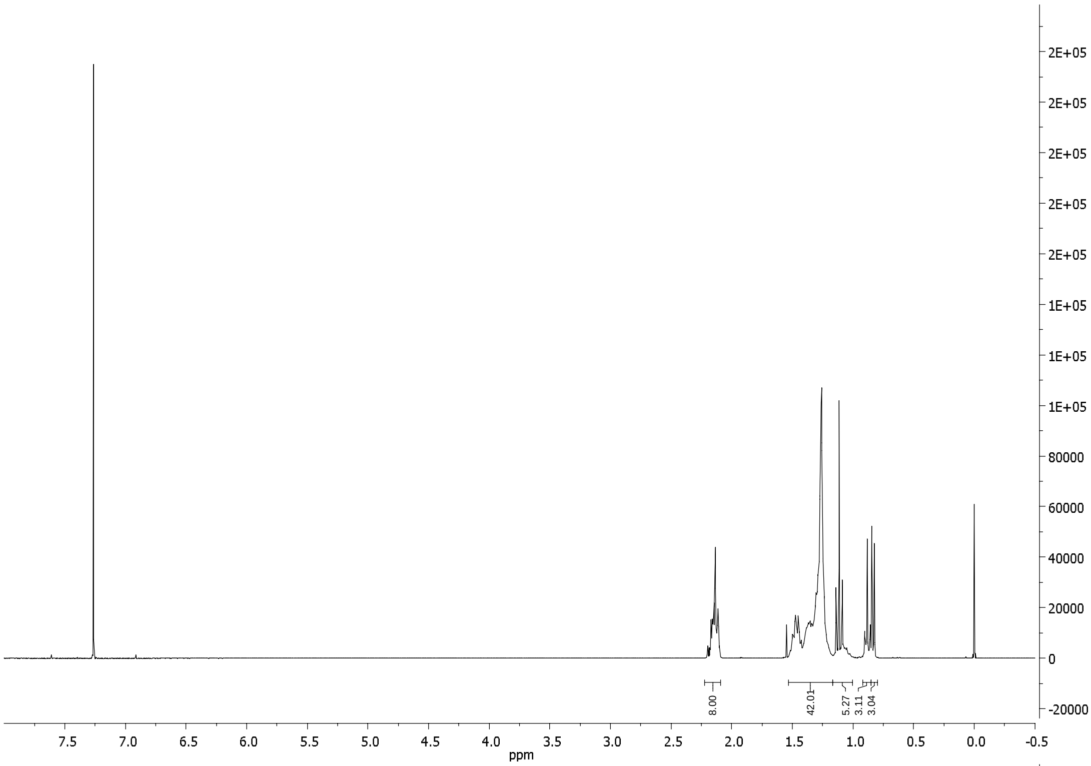

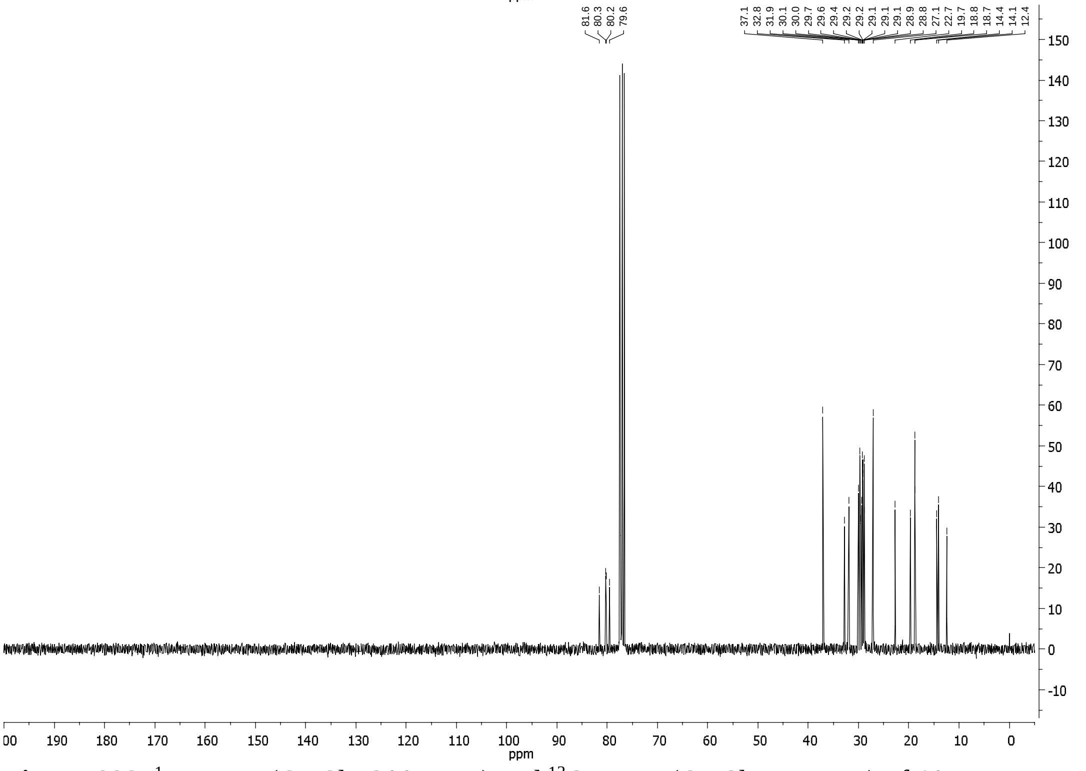

Figure S23. ^{1}H-NMR ($CDCl_3$, 300 MHz) and ^{13}C-NMR ($CDCl_3$, 75 MHz) of **10**.

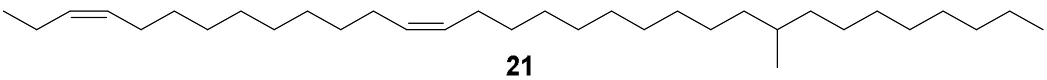

21

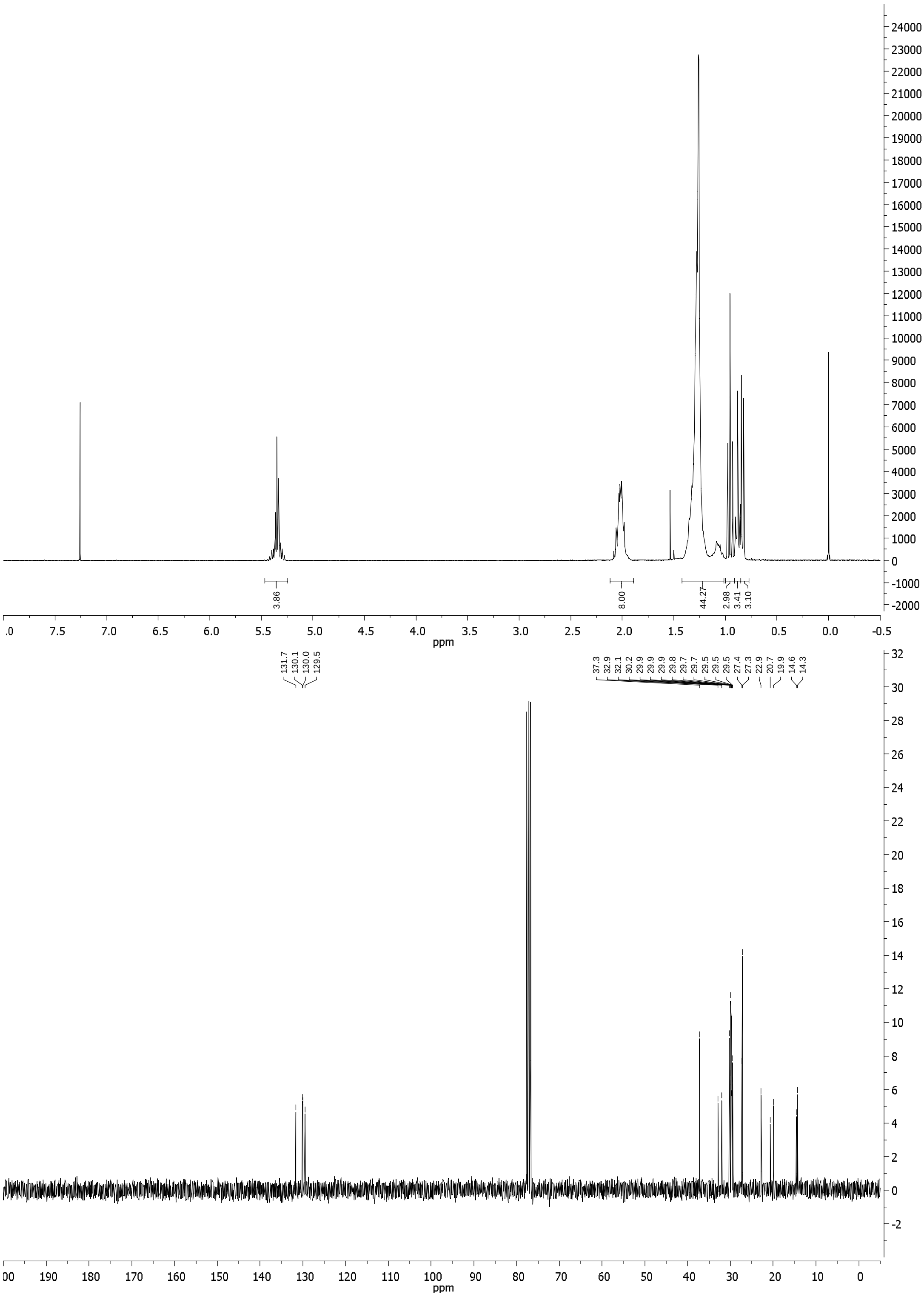

Figure S24. ^{1}H-NMR ($CDCl_3$, 300 MHz) and ^{13}C-NMR ($CDCl_3$, 75 MHz) of **21**.

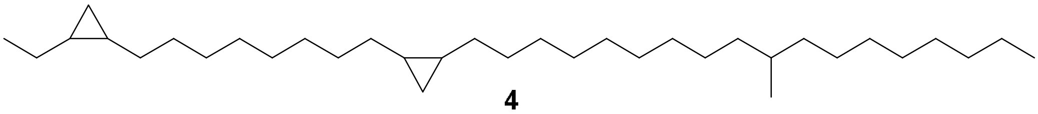

4

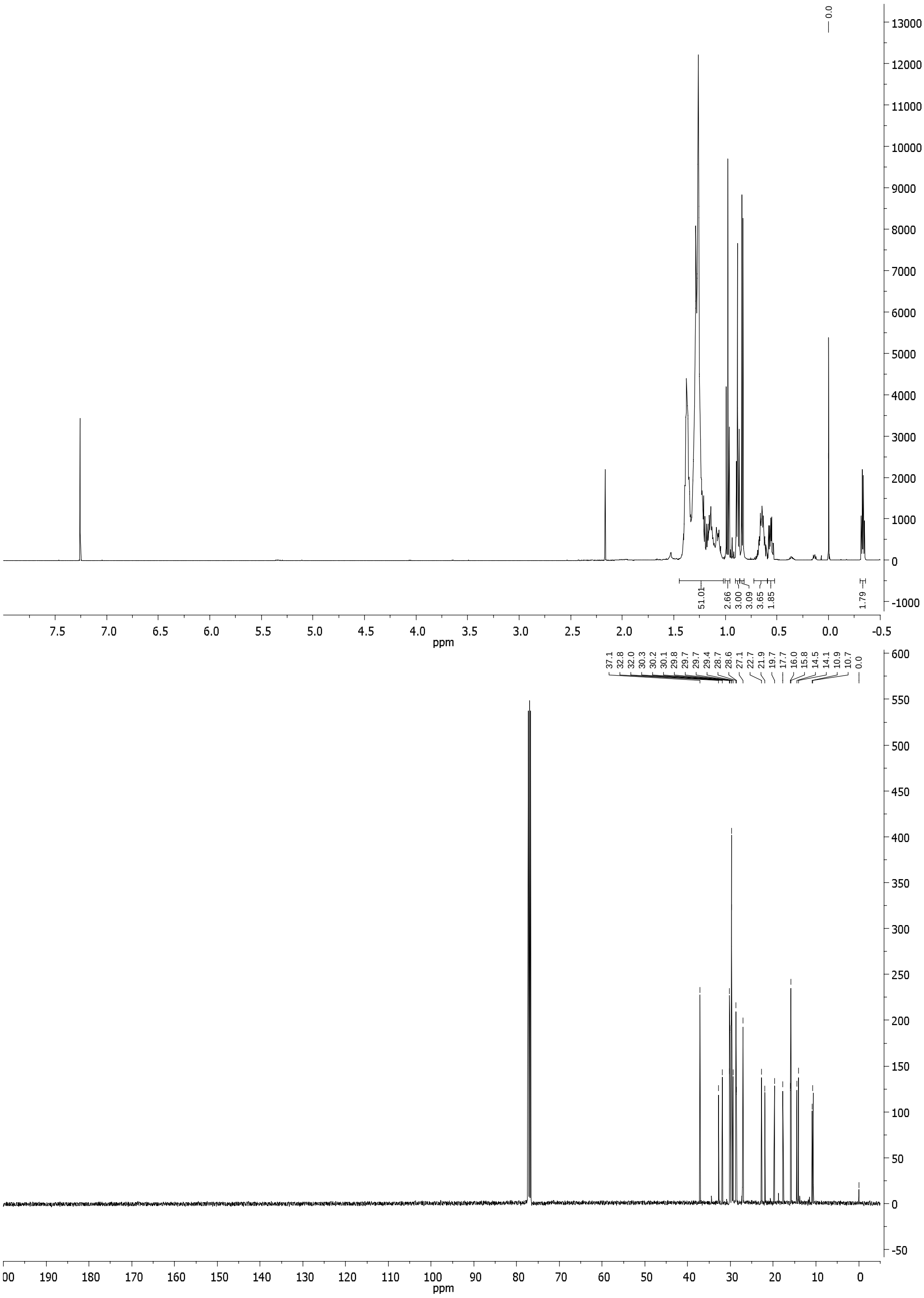

Figure S24. ^{1}H-NMR ($CDCl_3$, 500 MHz) and ^{13}C-NMR ($CDCl_3$, 125 MHz) of **4**.

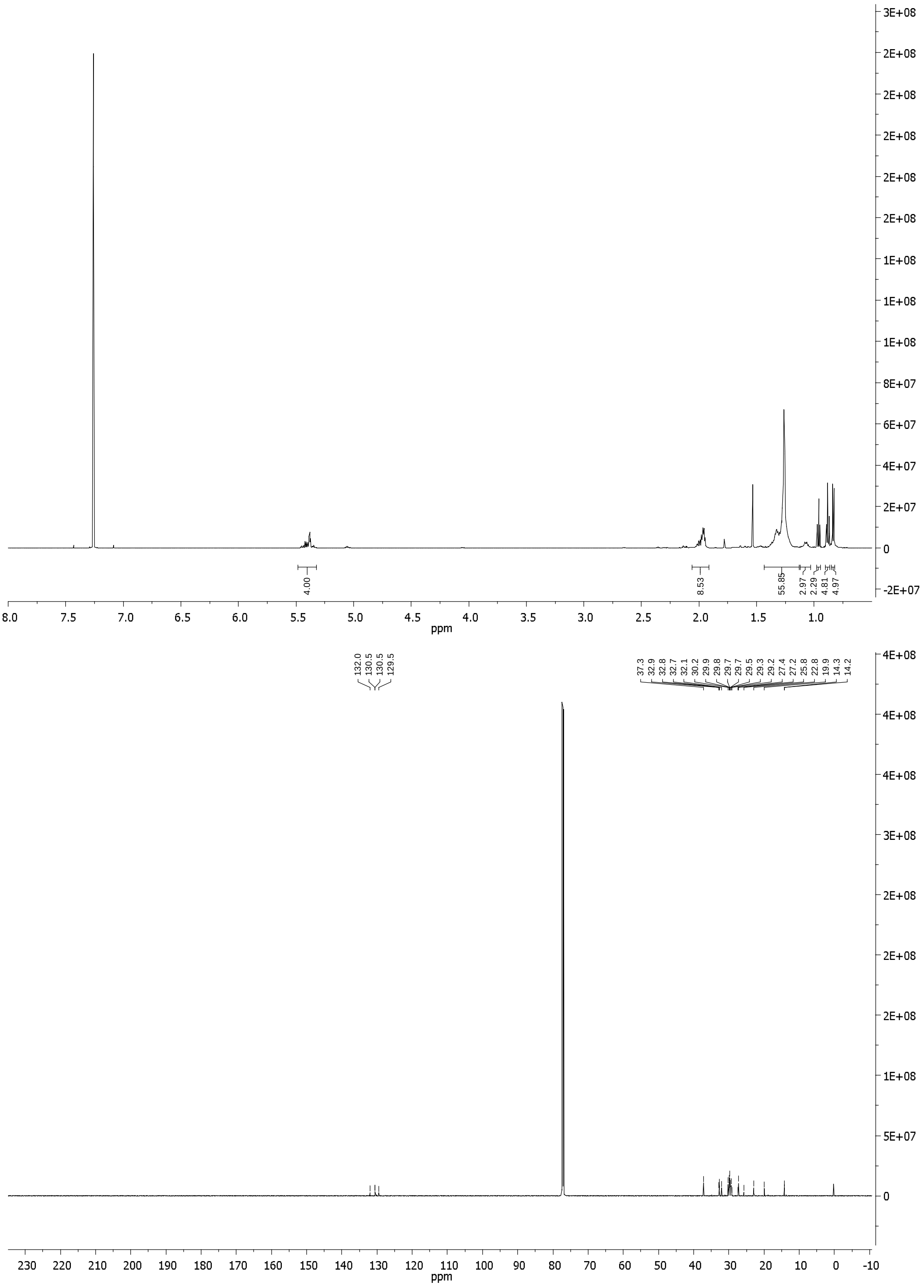

Figure S25. ^{1}H-NMR ($CDCl_3$, 600 MHz) and ^{13}C-NMR ($CDCl_3$, 150 MHz) of **22**.
